# Exosomes as Promising Therapeutic Tools for Regenerative Endodontic Therapy

**DOI:** 10.3390/biom14030330

**Published:** 2024-03-11

**Authors:** Qingyue Kong, Yujie Wang, Nan Jiang, Yifan Wang, Rui Wang, Xiaohan Hu, Jing Mao, Xin Shi

**Affiliations:** 1 Center of Stomatology, Tongji Hospital, Tongji Medical College, Huazhong University of Science and Technology, Wuhan 430030, China; u202013745@hust.edu.cn (Q.K.); m202176224@hust.edu.cn (Y.W.); d202181922@hust.edu.cn (Y.W.); u202213388@hust.edu.cn (R.W.); 2 School of Stomatology, Tongji Medical College, Huazhong University of Science and Technology, Wuhan 430030, China; 3 Hubei Province Key Laboratory of Oral and Maxillofacial Development and Regeneration, Wuhan 430022, China; 4 Central Laboratory, National Engineering Laboratory for Digital and Material Technology of Stomatology, Beijing Key Laboratory of Digital Stomatology, Peking University School and Hospital of Stomatology, Beijing 100081, China; nanjiang@bjmu.edu.cn; 5 Outpatient Department Office, Tongji Hospital, Tongji Medical College, Huazhong University of Science and Technology, Wuhan 430030, China; huxiaohan@tjh.tjmu.edu.cn

**Keywords:** pulpitis, regenerative endodontic therapy, pulp–dentin complex regeneration, stem cells, exosomes

## Abstract

Pulpitis is a common and frequent disease in dental clinics. Although vital pulp therapy and root canal treatment can stop the progression of inflammation, they do not allow for genuine structural regeneration and functional reconstruction of the pulp–dentin complex. In recent years, with the development of tissue engineering and regenerative medicine, research on stem cell-based regenerative endodontic therapy (RET) has achieved satisfactory preliminary results, significantly enhancing its clinical translational prospects. As one of the crucial paracrine effectors, the roles and functions of exosomes in pulp–dentin complex regeneration have gained considerable attention. Due to their advantages of cost-effectiveness, extensive sources, favorable biocompatibility, and high safety, exosomes are considered promising therapeutic tools to promote dental pulp regeneration. Accordingly, in this article, we first focus on the biological properties of exosomes, including their biogenesis, uptake, isolation, and characterization. Then, from the perspectives of cell proliferation, migration, odontogenesis, angiogenesis, and neurogenesis, we aim to reveal the roles and mechanisms of exosomes involved in regenerative endodontics. Lastly, immense efforts are made to illustrate the clinical strategies and influencing factors of exosomes applied in dental pulp regeneration, such as types of parental cells, culture conditions of parent cells, exosome concentrations, and scaffold materials, in an attempt to lay a solid foundation for exploring and facilitating the therapeutic strategy of exosome-based regenerative endodontic procedures.

## 1. Introduction

As the only soft tissue in the teeth, dental pulp is a highly vascularized and richly innervated loose connective tissue that is capable of transmitting temperature, mechanical stimuli, and chemical stimuli. It also possesses functions related to nutrition, protection, regeneration, and repair. Dental caries, trauma, periodontitis, and iatrogenic factors can lead to the occurrence of pulpitis, which ultimately triggers pulp necrosis if not promptly treated [1]. Note that the necrosis of dental pulp tissue will result in the loss of tooth vitality, and the vitality of the dental pulp is an essential factor determining the clinical strategies and prognosis of endodontic treatment [2].

For teeth affected by reversible pulpitis, where the apical part of the dental pulp remains active, vital pulp preservation is the preferred treatment strategy. Aimed at maintaining the vitality of dental pulp, vital pulp therapy induces pulp tissue recovery and reconstructs the protective mineralized barrier, thereby prolonging the longevity of the affected teeth [3]. Commonly applied approaches for pulp preservation in clinical settings include direct pulp capping and indirect pulp capping, where the capping materials are placed directly on the exposed pulp (direct pulp capping) or the dentin surface adjacent to the pulp (indirect pulp capping) in an attempt to preserve pulp vitality while inducing pulp tissue repair and tertiary dentin formation [4]. Therefore, pulp-capping materials should have favorable biocompatibility and adequate sealing properties. Currently, calcium hydroxide cement and mineral trioxide aggregates are frequently utilized as pulp-capping materials under clinical conditions [5], but both of them have undesirable shortcomings. In particular, calcium hydroxide will gradually dissolve after long-term application, leading to a higher possibility of microleakage and root canal reinfection [6,7]. On the other hand, mineral trioxide aggregates face multiple challenges, including long clinical setting times, insufficient antibacterial activity against specialized anaerobic bacteria, poor dentin bonding, and potential tooth discoloration [8]. It is worth emphasizing that when reversible pulpitis progresses into acute and chronic irreversible pulpitis, pulp tissue will not be able to heal itself due to anatomical constraints such as a narrow apical foramen and may further deteriorate into pulp necrosis or periapical disease [9]. At present, the first-selected treatment strategy for dental pulp necrosis is root canal treatment, which involves root canal preparation, root canal disinfection, and sealing of canal space with inert materials [10]. Even though root canal treatment can control root canal infection to a certain extent, effectively preserving dentin and extending the lifespan of the affected teeth, it results in the permanent loss of tooth vitality, sensation, and immune defense. In addition, compared to healthy teeth, teeth receiving root canal treatment exhibit considerably increased fragility, which makes them more susceptible to fractures or even tooth loss [11]. Furthermore, it should be mentioned that the root growth and development of immature permanent teeth in adolescents will stagnate after root canal treatment, leading to the failure of apical closure, which adversely compromises the long-term preservation and normal functions of the treated teeth [12]. In summary, the traditional treatment strategies for pulpitis in clinical practice are not ideal because they are unable to restore the complete structures and functions of the pulp–dentin complex. Therefore, adequately maintaining pulp vitality and promoting structural regeneration and functional reconstruction of the pulp–dentin complex has become an urgent bottleneck in endodontic therapy.

In recent years, with the rapid development of tissue engineering and stem cell medicine, RET has been recognized as the most promising strategy for treating pulpitis, which holds great promise for replacing traditional root canal treatment. As dental pulp stem cells (DPSCs), stem cells from human exfoliated deciduous teeth (SHED), stem cells from the apical papilla (SCAP), and other dental mesenchymal stem cells (MSCs) have been successively discovered, accumulating research has indicated that MSC transplantation can effectively promote pulp-like tissue regeneration in vivo [13,14]. Huang et al. confirmed that DPSCs and SCAP, when incorporated in root canals and implanted subcutaneously in mice, could induce the formation of pulp-like tissue accompanied by the deposition of new dentin [15]. Additionally, Xuan et al. used DPSC aggregates to successfully regenerate pulp-like tissues that structurally and functionally resembled original dental pulp, including layers of odontoblast-like cells, blood vessels, neural tissues, and positive responses to electrical stimulation [16]. However, the clinical translation of stem cell transplantation is confronted with various limitations in terms of stem cell sources, isolation and storage, safety, and medical ethics. With an in-depth understanding of the mechanisms regulating tissue regeneration and repair by stem cells, researchers have elucidated the crucial paracrine effects of MSCs on dental pulp regeneration. Reportedly, conditioned media (CM) from bone marrow-derived MSCs (BMMSCs), adipose-derived MSCs (AMSCs), and dental MSCs promote angiogenesis and induce odontoblastic differentiation of DPSCs [17,18,19]. Further studies have illuminated that the role of MSCs in promoting angiogenesis and inducing tissue regeneration primarily depends on the multifaceted functions of exosomes [20,21,22]. Consequently, exploring novel strategies for dental pulp regeneration without stem cell transplantation has become a research hotspot in regenerative endodontics, and an increasing number of endeavors are devoted to applying exosomes to enhance the regenerative potential of the pulp–dentin complex.

Exosomes are nanoscale extracellular vesicles secreted by nearly all cells and serve as critical mediators of intercellular communication [23]. Given the crucial roles of exosomes in transporting various biomolecules, including nucleic acids, proteins, and lipids, their potential applications in regenerative medicine have received widespread attention [24]. Recently, a large number of studies have documented that exosomes could enhance the proliferation and migration of dental stem cells and promote neural repair and vasculature formation, which ultimately contributes to the generation of pulp-like tissue, indicating that exosomes have a broad application prospect in RET [19,24,25,26]. In summary, concentrating on exosome-based regenerative endodontic treatment, this article first introduces the biological properties of exosomes, including their biogenesis process, internalization pathways, isolation and purification methods, and characterization techniques. Subsequently, considering the biological characteristics of dental pulp, we conduct an in-depth investigation into the feasibility and applicability of using exosomes for pulp–dentin complex regeneration. Furthermore, the roles and mechanisms by which exosomes regulate dental pulp regeneration are elucidated. Finally, we focus on discussing the clinical strategies and influencing factors of exosome application in RET in order to lay a solid foundation for developing exosome-induced regenerative endodontic strategies and facilitating their clinical translation, thereby promoting public oral health.

## 2. Biological Characteristics of Exosomes

**Figure 1:**
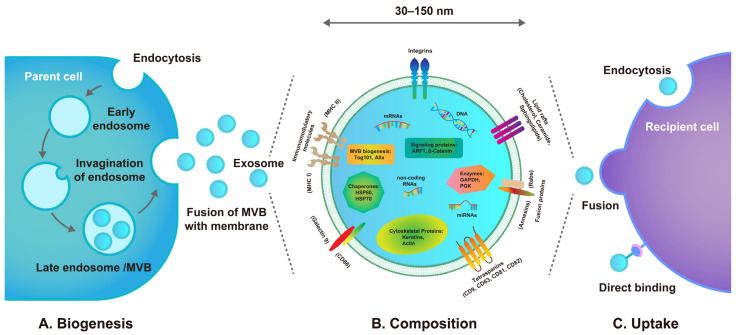
Schematic diagram of exosome biogenesis, composition, and uptake. (**A**) The biogenesis of exosomes starts with the invagination of the cytoplasmic membrane, resulting in the generation of early endosomes. Subsequently, the invagination of the endosomal membrane promotes the maturation of early endosomes into late endosomes, which contribute to the production of MVBs. Eventually, MVBs fuse with the cytoplasmic membrane, and exosomes are thus released into the extracellular environment. (**B**) Exosomes are lipid bilayer nanoparticles measuring 30–150 nm in diameter. They contain a variety of proteins, lipids, and nucleic acids, which are reflective of parental cells and can be delivered to recipient cells. (**C**) Exosomes can be internalized by recipient cells through endocytosis, fusion with the plasma membrane, or direct binding to the surface proteins.

As early as 1983, Pan and Johnstone pioneered the identification of a unique vesicular structure in the maturation process of sheep reticulocytes, which mediated the secretion of intracellular transferrin receptor protein to the extracellular compartment [27]. Subsequently, Johnstone et al. named this vesicular structure secreted from cells into the extracellular environment as exosomes [28]. Exosomes are nanoscale particles composed of phospholipid bilayers with a diameter of approximately 30–150 nm (Figure 1). They can carry a wide variety of bioactive substances, such as lipids, nucleic acids (mRNA, miRNA, etc.), cytoplasmic proteins, and transmembrane proteins, and display a characteristic cup-shaped structure under transmission electron microscopy [29]. Exosomes are derived from a diverse range of cell types, and almost all cells have the capability to secrete them via the exocytotic process [23]. Moreover, exosomes are extensively present in body fluids such as plasma, serum, urine, and saliva.

Exosomes belong to a subtype of extracellular vesicles that originate from the endosomal system [30] (Figure 1). Initially, the cytoplasmic membrane buds inward to form early endosomes [31]. Then, under the control of the endosomal sorting complexes required to transport proteins, the inward invagination of the endosomal membrane creates intraluminal vesicles (ILVs) within early endosomes, during which cytosolic proteins or nucleic acids are encapsulated into ILVs. The resulting ILVs facilitate the maturation of early endosomes into late endosomes, which can also be termed multivesicular bodies (MVBs). Finally, MVBs can fuse with the cytoplasmic membrane and induce the secretion of ILVs, which indicates that exosomes are consequently released into the extracellular environment. Exosomes regulate intercellular signaling primarily through paracrine actions and play crucial roles in various pathophysiological processes, such as cell proliferation, apoptosis, antigen presentation, immune modulation, tissue repair and regeneration, and tumorigenesis. It is now generally acknowledged that exosomes exert their biological effects on target cells through three distinct pathways: direct binding, fusion with the plasma membrane, and endocytosis [30,32] (Figure 1). When exosomes directly bind to membrane receptors on the recipient cells through exosomal surface proteins, intracellular signal transduction is activated, thereby regulating a series of biological behaviors. The fusion of exosomes with the plasma membrane of target cells leads to the non-selective delivery of exosomal contents into the cytoplasm, which represents the most efficient route for biomolecule transport. However, the most dominant pathway documented for exosome uptake is endocytosis, where intact exosomes are internalized and bound by the plasma membrane, subsequently fusing with the endosome system for cargo release.

The isolation of exosomes is a challenging task due to the heterogeneity in particle size, composition, function, and origin. Most of the currently available isolation techniques are unable to completely separate exosomes from lipoproteins with similar biophysical properties or extracellular vesicles derived from non-endosomal pathways, consequently resulting in low purity of exosomes. Therefore, efficient isolation and purification of exosomes remain a significant problem in the field of exosome research. At present, there are a variety of methods for exosome separation, each of which possesses its own advantages and disadvantages. As the gold standard for exosome extraction and isolation, ultracentrifugation is the most widely applied separation technique, primarily based on differences in the size and density of various components in the solution to obtain the desired components. Specifically, ultracentrifugation is suitable for separating large-dose samples with significantly different sedimentation coefficients [33]. Density gradient centrifugation is aimed at purifying exosomes and is commonly employed in combination with ultracentrifugation to improve the purity of exosomes. The polymer precipitation approach typically utilizes polyethylene glycol to reduce exosome solubility and then obtain exosomes through centrifugation. Polymer precipitation is relatively simple to perform and has a short analysis time, making it suitable for processing large samples. However, the purity and recovery rate of exosomes obtained through this method are low. Based on particle size, ultrafiltration and size-exclusion chromatography can distinguish exosomes from other components in the sample. Although these techniques are cost-effective and do not affect the biological characteristics of the obtained exosomes, the purity of the exosomes is diminished. Immunoaffinity chromatography is designed on the basis of specific binding between antibodies and ligands to separate the desired substances from heterogeneous mixtures. This technique can be used for qualitative and quantitative determination of exosomes, exhibiting the advantages of higher specificity, greater sensitivity, excellent purity, and superior yield. Notably, the preservation conditions for exosomes obtained via this method are relatively harsh. Microfluidic separation utilizes the physical and biochemical properties of dimensionally dense bodies for microscale separation, detection, and analysis, which is fast and requires only small amounts of samples and reagents.

The International Society for Extracellular Vesicles has proposed two proteins that need to be discriminated from exosomes, namely, transmembrane- or glycosylphosphatidylinositol-anchored proteins associated with plasma membranes and endosomes and cytosolic proteins recovered from extracellular vesicles. Thus, a combination of several characterization methods is warranted for consistently determining whether the extracted components are true exosomes [34,35]. Typically, the characterization of isolated exosomes should be carried out from three perspectives. Transmission electron microscopy has been extensively applied for the morphological characterization of exosomes, which are visualized as cup-shaped membrane structures. Nanoparticle tracking analysis allows the concentration and size distribution of exosomes to be detected, while Western blotting can examine exosomal-specific proteins, such as the tetraspanins CD9, CD63, and CD81 and the cytosolic proteins TSG101 and Alix. In addition, the ratio of nano-vesicle, nano-flow cytometry, and ultra-high-performance liquid chromatography can be employed to evaluate the purity of exosomes. Colorimetric or fluorescence analysis can assess the total amount of proteins. Significantly, quantitative estimation of specific molecules can be achieved by applying a sulfo-phospho-vanillin lipid assay, attenuated total reflectance Fourier transform infrared spectroscopy, enzyme-linked immunosorbent assay, and cytometric bead array.

## 3. The Role and Mechanism of Exosomes in Regulating Pulp–Dentin Complex Regeneration

In recent years, a large number of studies have demonstrated that exosomes are capable of inducing tissue and organ repair and regeneration in animal models of myocardial ischemia–reperfusion injury [36,37], brain injury [38,39], skin trauma [40], liver injury [41,42], and bone [43,44] and cartilage injuries [45,46], indicating that exosomes have enormous application potential in tissue engineering and regenerative medicine [47]. Research on the application of exosomes for dental pulp regeneration can be traced back to 2016, when Huang et al. reported that exosomes isolated from DPSCs undergoing odontogenic differentiation (DPSC-Od-Exos) significantly promoted odontoblastic differentiation of DPSCs in vitro and could induce the formation of pulp-like tissue that robustly expressed odontoblast-related proteins in vivo, confirming for the first time the feasibility of using exosomes for dental pulp regeneration [24]. Subsequently, immense efforts have been devoted to exploring exosome-based pulp–dentin complex regeneration, and a series of promising results have been achieved.

### 3.1. Exosomes in Cellular Proliferation and Migration

RET is dedicated to the structural regeneration and functional reconstruction of the pulp–dentin complex, which involves multiple biological procedures, including stem cell migration and proliferation, pulp revascularization and reinnervation, and dentin formation [48]. The migration and proliferation of stem cells initiate the regeneration and repair of pulp tissue. When pulp tissue is damaged, MSCs such as DPSCs and SCAP in the remaining healthy tissue will migrate to the pulp defect and rapidly proliferate to replenish the loss of stem cells. Studies have confirmed that exosomes can promote the migration and proliferation of dental-derived stem cells [49]. As revealed by Ivica et al., exosomes secreted by pulp tissue considerably enhance the proliferation and migration of BMMSCs, suggesting that exosomes can recruit endogenous BMMSCs from alveolar bone at the apex to the root canal space for pulp regeneration [50]. Further research has shown that exosomes derived from DPSCs (DPSC-Exos) could promote the migration of DPSCs in a dose-dependent manner, confirming the essential role of DPSC-Exos in improving the homing potential of DPSCs in the remaining healthy pulp tissue and inducing endogenous pulp regeneration. Notably, previous studies have elucidated that, on the basis of exosomes, Schwann cells originating from the neural crest could cooperate with DPSCs to regulate the regeneration and repair of the pulp–dentin complex [51,52]. On the one hand, exosomes derived from Schwann cells promote the proliferation and multilineage differentiation of human dental pulp cells [51]. On the other hand, it has been recently evidenced by the CCK-8 assay and the Transwell migration assay that DPSC-Exos could positively regulate the proliferation and migration of Schwann cells [52]. Consequently, the positive feedback mechanism between Schwann cells and DPSCs through exosomes further establishes the basis for enhancing the biological properties of DPSCs.

### 3.2. Exosomes in Odontogenesis

To achieve structurally and functionally complete pulp–dentin complex regeneration, it is crucial to initiate the multilineage differentiation potential of stem cells. When MSCs are recruited or implanted into the site of pulp injury, one of their primary missions is to differentiate into odontoblasts and form tubular dentin to repair damaged tooth hard tissue, laying the foundation for rebuilding the protective barrier of dental pulp and restoring the biological functions of the pulp–dentin complex. Therefore, inducing the odontogenic differentiation of MSCs has become one of the critical tasks for pulp–dentin complex regeneration. Multiple studies have documented that exosomes could promote the multidirectional differentiation of stem cells, thus endowing them with promising prospects for dental pulp regeneration [53]. Previously, Hu et al. revealed in vitro that DPSC-Od-Exos could promote the odontogenic differentiation of DPSCs through the TGFβ1/Smads signaling pathway [54]. Consistent with the above results, Swanson et al. indicated that DPSC-Exos could induce the differentiation of DPSCs into odontoblast-like cells in vitro by activating MAPK signaling, remarkably promoting the expression of odontogenic-related genes such as *dentin sialophosphoprotein (DSPP)*, *bone sialoprotein*, and *vascular endothelial growth factor (VEGF)* while increasing the accumulation of orange-red mineral nodules as assessed by alizarin red staining [55]. Additionally, in vivo experiments have shown that DPSC-Exos considerably enhance the reconstruction of new tubular dentin and increase the number of odontoblast-like cells. It is worth noting that DPSC-Exos has also been reported to enormously facilitate the mRNA expression levels of odontogenic markers in Schwann cells, including *DSPP*, *dentin matrix protein 1 (DMP1)*, *osteocalcin (OCN)*, and *Runt-related transcription factor 2 (RUNX2)*, and markedly promote the deposition of red-brown calcium nodules [52]. Taken together, the above results indicate that DPSC-Exos hold immense potential to improve the odontogenic competency of Schwann cells and DPSCs. However, the specific contents delivered by DPSC-Exos remain to be elucidated in the future. In addition to DPSC-Exos, Zhuang et al. identified that under osteogenic induction conditions, exosomes derived from SCAP (SCAP-Exos) significantly upregulated gene and protein expression levels of DSPP in BMMSCs [56]. SCAP-Exos and BMMSCs were then filled into tooth fragments and implanted subcutaneously in mice for 12 weeks. As detected by histological analysis, a layer of new continuous dentin was generated. Moreover, compared with the control group without SCAP-Exos implantation, the thickness of the newly formed dentin layer was substantially augmented, and the number of polarized odontoblasts with high columnar morphology was also considerably elevated, further confirming that SCAP-Exos are capable of enhancing the odontogenic differentiation and dentin formation of BMMSCs, thereby contributing to the regeneration of the pulp–dentine complex.

### 3.3. Exosomes in Angiogenesis

**Figure 2:**
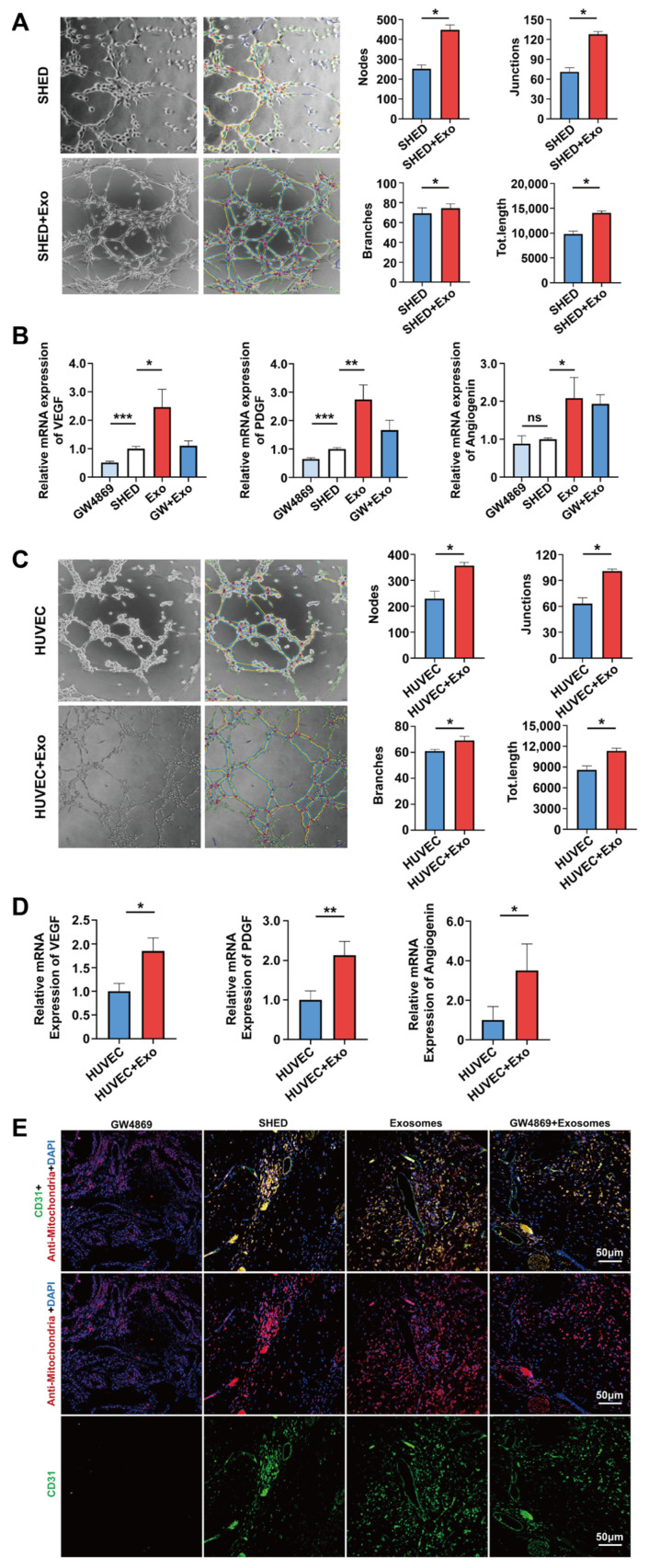
SA-Exos enhance tubular formation in vitro and pulp angiogenesis in vivo. (**A**,**B**) SA-Exos stimulated endothelial differentiation of SHED. (**A**) As indicated by the tube formation assay, SA-Exos exhibited a promoting effect on the tubular structure formation of SHED, which was further confirmed by a considerably enhanced number of nodes, connections, branches, and total length. (**B**) Real-time quantitative polymerase chain reaction (RT-qPCR) demonstrated that SA-Exo significantly upregulated the expression of angiogenic-related genes, including *VEGF*, *Ang*, and *PDGF*, in SHED. However, the application of GW4869, which inhibited exosome secretion in SHED, resulted in the downregulation of *VEGF* and *PDGF* expression, which could be partially alleviated by complementing SA-Exo. * *p* < 0.05; ** *p* < 0.01; *** *p* < 0.001; ns: No significant differences. (**C**,**D**) SA-Exos enhanced the angiogenic potential of HUVECs. (**C**) SA-Exos substantially facilitated the formation of vascular-like networks in HUVECs, as evidenced by the increased nodes, connections, branches, and total length. (**D**) Consistently, mRNA expression of the vascular markers *VEGF*, *Ang*, and *PDGF* was remarkably promoted in HUVEC after co-culture with SA-Exos. * *p* < 0.05; ** *p* < 0.01. (**E**) Immunofluorescence staining for CD31 verified that subcutaneous transplantation of SHED aggregates combined with SA-Exos (the third column) in mice markedly enhanced the generation of CD31^+^ blood vessels compared with the SHED aggregate group (the second column). Notably, in contrast with the GW4869 pretreatment group (the left-most column), which inhibited exosome secretion of SHED aggregates, the exogenous supplementation with SA-Exos (the right-most column) contributed to a higher intensity of green fluorescence, suggesting that SA-Exos rescued the angiogenic capability of SHED aggregates. Scale bar is shown. Reproduced with permission [60].

Enriched vascular networks can provide sufficient oxygen and nutrient supply to the regenerated dental pulp and excrete metabolic products such as carbon dioxide. Therefore, angiogenesis is a crucial factor determining the success of pulp–dentin complex regeneration and represents a prerequisite for restoring the normal physiological functions of pulp tissue. Existing research has reported that DPSC-Exos could induce the proliferation of human umbilical vein endothelial cells (HUVECs) in vitro and promote the formation of vascular-like tubular structures, as evidenced by the obviously increased number of loops and nodes and length of tubes as well as upregulated expression of angiogenesis-related genes and proteins, such as VEGF and angiopoietin-2 (Ang-2), laying the foundation for promoting vascular formation and dental pulp functional recovery in vivo [57]. According to Zhang et al., after the application of the inhibitor GW4869 in PDLSCs, which diminished the secretion of exosomes, the tubular structure formation capability of HUVECs co-cultured with PDLSCs was significantly suppressed, accompanied by a remarkably decreased number of nets, loops, and branching points as well as the length of tubes [58]. Furthermore, the expression of the angiogenesis markers CD31 and VEGFA was substantially attenuated, proving that PDLSCs can promote the angiogenesis of HUVECs through the exosome-mediated pathway. Xian et al. co-cultured dental pulp cell-derived exosomes (DPC-Exos) with HUVECs and observed that the formation of vascular networks and the length of tubes were considerably enhanced [59]. Correspondingly, further investigation revealed that the mRNA levels of *VEGFA*, *vascular endothelial growth factor receptor 2 (VEGFR2)*, and *matrix metalloproteinase-9 (MMP-9)* as well as the protein expression levels of VEGFA, MMP-9, and fibroblast growth factor-2 (FGF-2) were remarkably upregulated. In terms of underlying molecular mechanisms, this study disclosed that the administration of DPC-Exos markedly strengthened the phosphorylation of p38 MAPK in HUVECs, thus promoting angiogenesis by activating the p38 MAPK signaling pathway. As per a recent report by Wu et al., exosomes derived from SHED aggregates (SA-Exos) significantly facilitated the tubular length and the number of nodes, junctions, and branches of newly formed blood vessels in SHED and HUVECs as well as the expression of angiogenesis-related markers, including *VEGF*, *Ang*, and *platelet-derived growth factor (PDGF)*, indicating that SA-Exos can not only promote the angiogenic potential of HUVECs but also directly enhance the endothelial differentiation potential of SHED [60] (Figure 2). Regarding the underlying mechanisms, miRNA-26a delivered by SA-Exos was closely associated with improved angiogenesis in SHED and HUVECs by activating the TGF-β/SMAD2/3 signaling pathway. In addition, as illustrated by immunofluorescence staining, subcutaneous implantation of SHED aggregates combined with SA-Exos in mice substantially prompted the generation of CD31^+^ blood vessels (Figure 2). Intriguingly, as opposed to the GW4869-treated group, which inhibited CD31 expression, exogenously supplemented SA-Exos considerably rescued the fluorescence intensity of CD31, highlighting that SA-Exos can ameliorate the angiogenic capability of SHED aggregates. Collectively, these findings indicate that exosomes exert great promise for enhancing pulp revascularization, which is beneficial for the advancement of pulp–dentin complex regeneration.

### 3.4. Exosomes in Neurogenesis

The regeneration of pulp nerves and the restoration of sensory function are essential for completely reestablishing the pulp–dentin complex. DPSCs and SHED, originating from the cranial neural crest, are precursor cells for the development of neural tissues [61]. Consequently, in comparison with MSCs from other sources, these dental stem cells show tremendous potential for inducing dental pulp nerve regeneration. DPSCs have been verified to promote nerve regeneration through paracrine mechanisms by secreting nerve growth factors, brain-derived neurotrophic factor (BDNF), and glial cell line-derived neurotrophic factor, thus demonstrating favorable neuroprotective effects [62]. Therefore, it is reasonable to believe that DPSC-Exos have the ability to promote nerve regeneration and neurological function recovery. As indicated by Venugopal et al., DPSC-Exos exhibited desirable neuroprotective effects against kainic acid-induced excitotoxicity in vitro [63]. Further studies have clarified that DPSC-Exos could initiate anti-apoptotic and anti-necrotic mechanisms, upregulate the expression of BDNF and the apoptosis inhibitory factor Bcl-2 in neurons, and prevent cell apoptosis by activating the PI3K-Bcl-2 pathway. In addition, according to Mao et al., the local delivery of extracellular vesicles from gingiva-derived mesenchymal stem cells (GMSC-EVs) played a crucial role in promoting axonal regeneration and functional recovery of damaged sciatic nerves in mice [64]. At the same time, GMSC-Evs also facilitated the proliferation and migration of Schwann cells and significantly elevated genes and proteins of differentiation, myelination, or repair phenotypes of Schwann cells in vitro, such as early growth response 2 and glial fibrillary acidic protein. Furthermore, Jarmalaviciute et al. verified that exosomes derived from SHED could substantially repress the apoptosis of human dopaminergic neurons induced by neurotoxin 6-hydroxydopamine hydrochloride, the hidden mechanism of which was possibly related to the reduction of oxidative stress sensitivity of dopaminergic neurons [65]. Although there is a lack of studies directly correlated with the role and mechanism of exosomes in promoting dental pulp nerve regeneration, currently available experimental results suggest that exosomes secreted by dental stem cells possess enormous potential to restore pulp neurological function.

In conclusion, dental MSC-derived exosomes can ameliorate the migration, proliferation, and multilineage differentiation capabilities of stem cells, making them an ideal material to foster the regeneration of dentin, blood vessels, and nerves, which potentiate the development of RET.

## 4. Influencing Factors of Exosomes Regulating Pulp–Dentin Complex Regeneration

### 4.1. Types of Parent Cells

Exosomes derived from different parental cells carry specific bioactive molecules and exhibit unique biological functions that are closely related to the parent cells. It has been demonstrated that exosomes originating from osteoblasts contain osteogenic factors, which could promote the osteogenic differentiation of MSCs [66]. In contrast, exosomes derived from adipocytes could potentially trigger the adipogenic differentiation of MSCs. Hence, before applying exosomes to clinical endodontic regenerative practice, careful consideration must be given to the parental cell types from which the exosomes originate.

**Figure 3:**
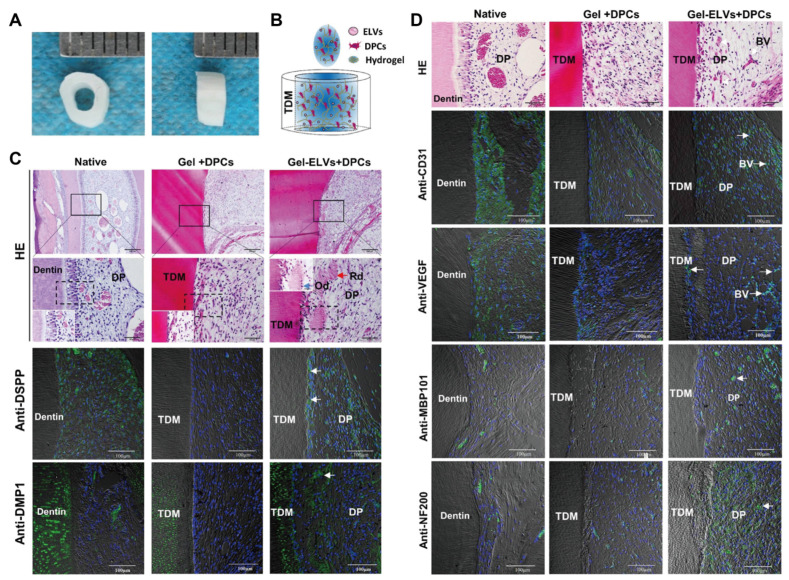
HERS-ELVs promote regeneration of the pulp–dentin complex in vivo. (**A**) The prepared dentin tubes. (**B**) Schematic illustration of subcutaneous transplants incorporating collagen gel embedded with DPCs and HERS-ELVs. (**C**,**D**) Multifaceted roles of HERS-ELVs in facilitating odontogenesis, angiogenesis, and neurogenesis. (**C**) As revealed by HE staining, newly formed pulp–dentin complex structures, including tubular dentin (red arrow), polarized odontoblast-like cells (blue arrow), and pulp-like soft tissue, could be identified in the Gel-ELVs + DPCs group. Immunofluorescence analysis indicated that Gel-ELVs + DPCs resulted in positive staining of the odontogenesis-associated proteins DSPP and DMP1 at the interface between pulp-like tissue and dentin. (**D**) HE staining also confirmed that the implantation of Gel-ELVs + DPCs led to enriched blood vessel formation (white arrows) in the pulp chambers. Concomitantly, immunofluorescence staining for angiogenic markers, including CD31 and VEGF, was considerably enhanced. Furthermore, the expression of the neurogenesis-related proteins MBP101 and NF200 (white arrows) was obviously observed in the Gel-ELVs + DPCs group. Scale bars: (**C**) first row: 200 µm, second row: 50 µm; (**D**) first row: 50 µm. TDM: treated dentin matrix; Rd: regenerated dentin-like tissue; Od: odontoblast-like cell; BV: blood vessels; DP: dental pulp-like tissue. Reproduced with permission [25].

As mentioned earlier, exosomes derived from DPSCs [54], SHED [60], and SCAP [56] have been broadly exploited to facilitate the regeneration of the pulp–dentin complex. DPSCs have strong self-renewal capacity and multidirectional differentiation potential [67,68] and can be isolated from the pulp tissue of extracted healthy wisdom teeth and orthodontic teeth [69]. According to Huang et al., when DPSC-Exos and DPSCs embedded in type I collagen membranes were encapsulated into root slices and implanted subcutaneously into the backs of nude mice, they not only induced the formation of reparative dentin but also boosted the generation of vascularized dental pulp-like tissue [24]. Moreover, immunohistochemistry staining and RT-qPCR confirmed that the expression levels of odontogenesis- and angiogenesis-related markers, such as DMP1, DSPP, RUNX2, TGF-β, and PDGF, were considerably augmented in the regenerated dental pulp-like tissue. Hence, DPSC-Exos elicited a conducive effect on the odontogenic differentiation and vascular regeneration of DPSCs. SHED, which can be isolated from the pulp of human deciduous teeth, has emerged as an essential MSC source for dental pulp regeneration due to its lower immunogenicity and fewer ethical issues when compared with DPSCs [70]. Guo et al. documented that the orthotopic implantation of SHED in the teeth of minipigs resulted in the regeneration of full-length dental pulp tissue, which contained the odontoblast layer, blood vessels, and nerves, as verified by HE staining and positive immunostaining of CD31 and neurofilament (NF) [71]. Using subcutaneous injection models, Yuan et al. reported that SHED encapsulated in injectable simvastatin-functionalized gelatin methacrylate (GelMA) cryogel microspheres was capable of regenerating vascularized pulp tissue in the presence of DMP1- and DSPP-positive odontoblast-like cells in intimate contact with the native dentin wall [72]. Taken together, these results preliminarily demonstrate the feasibility of SHED in stem cell-based regenerative endodontic dentistry. To illuminate the potency of the SHED secretome on pulp regeneration, Vu et al. recently depicted that SHED-CM exerted a promotive efficiency on the proliferation, migration, and odonto/osteogenic differentiation of DPSCs, raising the possibility that SHED-derived exosomes may hold promising applications for pulp regeneration [73]. Derived from the apical papilla of young immature permanent teeth, SCAP has a more pronounced potential for proliferation, migration, and odontogenic differentiation in comparison to DPSCs, thus expanding their possibility to stimulate pulp regeneration [74]. Na et al. discovered that, in sharp contrast with SCAP sheets, SCAP pellets were more prone to initiating odonto/osteogenic differentiation, as revealed by significantly higher mRNA expression levels of *DSPP*, *RUNX2*, *alkaline phosphatase* (*ALP*), and *bone sialoprotein* (*BSP*) [75]. In particular, after subcutaneous transplantation of SCAP pellets encapsulated in root canals for 6 weeks, highly vascularized pulp-like tissue with uniform cell density was observed, accompanied by continuous deposition of new tubular dentin around the original dentin walls. In addition, the polarized odontoblast-like cells expressing DSPP, ALP, and BSP proteins were detected to arrange on the newly formed dentin tissue and extend their cellular protrusions into the dentinal tubules. Together, these findings demonstrate that SCAP pellets possess outstanding odontogenic properties and display immense potential for facilitating pulp–dentin complex regeneration. In light of this, exosomes secreted by SCAP pellets may represent feasible and desirable bioactive substances to prompt the development of RET, which deserves particular attention. Hertwig’s epithelial root sheath (HERS) is a bilayered sheath-like structure comprising the inner and outer enamel epithelium. It has pivotal impacts on root development and periodontal tissue formation [76]. To gain insight into how HERS and dental papilla cells (DPCs) mediate pulp–dentin complex regeneration, Zhang et al. extracted exosome-like vesicles from HERS (HERS-ELVs) in a recent study and reported that HERS-ELVs at 80 μg/mL considerably enhanced the proliferative and migratory capacities of DPCs as well as their odontogenic potential by activating the Wnt/β-catenin signaling pathway [25]. Moreover, it was further elucidated in vitro that HERS-ELVs remarkably promoted the vascular formation of HUVECs, as suggested by the increased nodes, junctions, and meshes. Encouragingly, HERS-ELVs also exerted a boosting effect on the neurogenic differentiation of DPCs, significantly augmenting the expression of the neuronal marker proteins nestin and NF200. Aimed at comprehensively understanding the pleiotropic roles of HERS-ELVs in enhancing functional pulp–dentin complex regeneration, the research group subcutaneously transplanted DPCs and HERS-ELVs incorporated in collagen gel into nude mice. Four weeks later, newly formed pulp–dentin complex structures, including tubular dentin, polarized odontoblast-like cells, and blood vessel-enriched pulp-like soft tissue, could be observed under HE staining (Figure 3). Furthermore, immunostaining demonstrated a markedly increased expression of DSPP and DMP1 at the interface between pulp-like tissue and dentin (Figure 3). Meanwhile, the angiogenic markers CD31 and VEGF and the neurogenesis-related proteins MBP101 and NF200 were obviously identified within the pulp chamber (Figure 3), indicating that HERS-ELVs were capable of fostering multilineage differentiation competency in DPCs. Collectively, these results reveal that dental-derived exosomes offer broad prospects for enhancing proliferation and migration as well as odontogenesis, angiogenesis, and neurogenesis, paving the way for promoting complete functional regeneration of the pulp–dentin complex.

Notably, the application of exosomes obtained from non-dental MSCs in regenerative endodontic procedures requires considerable consideration to ensure the desired therapeutic efficacy. It has been shown that exosomes derived from AMSCs (AMSC-Exos) mainly play critical roles in inducing osteogenic differentiation, inhibiting osteocyte apoptosis, and regulating inflammation [77,78,79]. According to Huang et al., AMSC-Exos not only promoted the proliferation and migration of BMMSCs but also facilitated ALP activity and gene expression of *ALP* and *RUNX2*, thus reinforcing the osteogenic potential of BMMSCs [77]. Additionally, Song et al. further clarified that AMSC-Exos could significantly inhibit apoptosis of bone cells under hypoxic–ischemic conditions [78]. Moreover, AMSC-Exos have been demonstrated to drive M2 macrophage polarization, alleviate inflammatory responses, and induce beiging of white adipose tissue, consequently preventing adipose tissue inflammation and metabolic disorders [79]. Therefore, the employment of AMSC-Exos in RET remains to be elucidated. Human umbilical cord mesenchymal stem cells (hUCMSCs) are attractive non-dental MSC candidates that exist in Wharton’s jelly of umbilical cords. They possess the advantages of convenient collection; non-invasive acquisition; high stem cell proportion; ease of amplification, storage, and transportation; and superior anti-inflammatory and immunoregulatory capabilities [80]. Multiple studies have proven that hUCMSCs potentially promote tissue regeneration and repair, suppress cell apoptosis, and modulate immune responses [81,82]. As documented by Chen et al., hUCMSCs cultured in tooth germ cell-conditioned medium could differentiate into odontoblast-like cells, which express the odontogenic markers DMP1 and dentin sialoprotein (DSP) [83]. More importantly, when hUCMSCs were subcutaneously implanted into the backs of nude mice with human dentin matrix for 8 weeks, new dentin-like hard tissue and odontoblast-like cells were observed, which were also immunohistochemically identified by positive expression of DMP1 and DSP. In a recent clinical randomized controlled trial conducted by Brizuela et al., hUCMSCs encapsulated in platelet-poor plasma were injected into the pulp cavities of mature permanent teeth that suffered from pulp necrosis and apical periodontitis [84]. As a consequence, after a 12-month follow-up, apical bone lesions were absent, and percussion pain had disappeared entirely. In addition, significant enhancement of pulp vitality, as shown by electric and thermal pulp tests, and the recovery of blood flow perfusion, as measured by laser doppler flowmetry, were validated, which provided insights into the safety and effectiveness of hUCMSCs for dental pulp regeneration therapy. Unfortunately, there is currently a lack of in-depth research on the role and prospect of exosomes derived from hUCMSCs (hUCMSC-Exos) in pulp–dentin complex regeneration, which warrants further investigation. It is worth emphasizing that the co-culture of hUCMSCs and human dental pulp cells gave rise to enormously promoted proliferation of the co-cultured cells compared to individually cultured hUCMSCs or human dental pulp cells [80]. Furthermore, mRNA expression levels of *DSPP*, *DMP1*, *ALP*, and *OCN* as well as ALP activity were considerably enhanced, accompanied by a remarkably elevated number and density of mineralized nodules, which confirmed that co-culturing hUCMSCs and hDPCs was beneficial for cell proliferative ability and odontoblastic differentiation competency. Therefore, although the mechanisms by which hUCMSCs induce dental pulp regeneration and the interaction and regulatory processes between hUCMSCs and human dental pulp cells remain to be elucidated, it is speculated that hUCMSCs-Exos have the potential to ameliorate the microenvironment of pulp–dentin regeneration and play pivotal roles in hUCMSC-mediated regenerative endodontic treatment.

In summary, the biological functions of exosomes are closely correlated with the type of donor cells from which they originate. Accordingly, when exploring the feasibility and applicability of exosomes for RET, researchers should prioritize dental-derived stem cells as the parental cells for exosome production, given they have a more substantial promoting effect on pulp–dentin complex regeneration.

### 4.2. Culture Environment of Parental Cells

Different culture environments, such as inflammatory stimulation, conditioned culture, and hypoxia induction, can have various effects on the parental cells from which exosomes are derived, and exosomes secreted by parental cells under diverse conditions possess specific bioactive components and biological functions. According to Li et al., pretreating DPSCs with 1 μg/mL lipopolysaccharides (LPS) could significantly increase their exosome secretion [52]. Further studies have also elaborated that exosomes derived from LPS-preconditioned DPSCs (DPSC-L-Exos) obviously facilitate the proliferation, migration, and odontoblastic differentiation of Schwann cells. Consistent with the above results, Zhang et al. confirmed that tumor necrosis factor-α (TNF-α) preconditioning could also markedly promote the secretion efficiency of exosomes from PDLSCs [58]. Notably, exosomes originating from TNF-α-preconditioned PDLSCs (PDLSC-T-Exos) exerted an enhancing impact on the vascular formation ability of HUVECs compared to normal exosomes, contributing to considerably increased total tube length, total branching points, total loops, covered area, and total nets. Regarding the hidden molecular mechanism, it was illustrated that the decreased expression of miRNA-17-5p in PDLSC-T-Exos resulted in upregulated VEGFA expression, which in turn promoted the angiogenic capability of HUVECs. Therefore, this study revealed that mild inflammatory preconditioning could substantially improve the proangiogenic ability of exosomes derived from PDLSCs. Meanwhile, by conducting transwell and CCK-8 assays, Huang et al. clarified that DPSC-L-Exos pretreated with 5 μg/mL LPS significantly prompted the migration and proliferation abilities of HUVECs [85]. Importantly, in comparison to normal exosomes, DPSC-L-Exos intervention led to more regularly organized vascular-like structures, as indicated by the increased total tube length and the number of junction points. Moreover, gene expression levels of proangiogenic factors such as VEGF and VEGFR2 were demonstrated to expedite as a consequence of DPSC-L-Exo application. In addition to improving the environment for the regeneration of pulp tissue, DPSC-Exos have also been elucidated to alleviate dental pulp inflammation by transferring higher levels of anti-inflammatory factors, such as TGF-β and interleukin-10 (IL-10) [86]. In conclusion, appropriate inflammatory stimulation not only facilitates the release of exosomes from stem cells but also remarkably enhances their functions, which permit exosomes to suppress pulp inflammatory response and induce pulp–dentin complex regeneration, thereby endowing them with broader clinical translation potential. In light of this, aside from LPS and TNF-α, the prospects of other low-concentration proinflammatory factors, such as interferon-γ, in promoting exosome functions remain to be illuminated in the future, and they are expected to open up new routes for developing exosome-based regenerative endodontic practice.

Focusing on the impact of conditioned culture on exosomes, Wang et al. reported that exosomes obtained from SHED undergoing osteogenic induction were able to promote osteogenic differentiation of PDLSCs, markedly augmenting ALP activity and upregulating the expression of the osteogenic-related genes *RUNX2*, *OCN*, and *osteopontin* [87]. By conducting miRNA sequencing analysis, Hu et al. verified that, compared with DPSC-Exos, the enrichment levels of multiple miRNAs in DPSC-Od-Exos were significantly altered, among which the expression level of miRNA-27a-5p was incredibly increased [54]. In addition, miRNA-27a-5p mimics could considerably enhance the protein expression levels of DSP, DMP1, ALP, and RUNX2, highlighting that DPSC-Od-Exos could potentially induce odontogenic differentiation of DPSCs by delivering miRNA-27a-5p. Apart from conditioned culture, three-dimensional (3D) cell culture also represents a desirable pretreatment strategy that can effectively facilitate dental pulp regeneration. Faruqu et al. observed that, in comparison with the two-dimensional culture environment, the multipotency and exosome secretion efficiency of DPSCs were further enhanced under 3D culture conditions [88]. As revealed by Zhang et al., SA-Exos obtained under 3D culture exhibited stronger multipotent differentiation capabilities and substantially promoted odontogenesis, angiogenesis, and neurogenesis, which successfully induced the regeneration of the pulp–dentin complex in vivo [89]. Reportedly, the newly regenerated pulp tissue displayed regular and uniform structures with the presence of abundant blood vessels and nerve distribution. Furthermore, a continuous layer of odontoblast-like cells was identified to arrange along the newly deposited dentin in a palisade-like manner. Intriguingly, hypoxic conditions have been elaborated to drive stem cell differentiation and angiogenic responses in various injured tissues, thus promoting tissue repair and regeneration [90]. Accumulating evidence supports the idea that hypoxia can considerably alter exosome contents, facilitate their production and uptake efficiency, and enhance their angiogenic potential [91,92]. In this regard, Li et al. discovered in vitro that exosomes derived from hypoxia-preconditioned DPSCs (DPSC-H-Exos) enormously strengthened the proliferation and migration capabilities of HUVECs compared to exosomes obtained from DPSCs under normoxia [57]. Furthermore, DPSC-H-Exos were more beneficial for the formation of tubular-like structures, as evidenced by the significantly increased total tube length and number of junctions and the remarkable upregulation of angiogenesis regulatory proteins, including VEGFA, CD31, and stromal cell-derived factor-1. Mechanically, by performing proteomics analysis, this study underlined that the addition of DPSC-H-Exos could partially rescue the inhibitory role of lysyl oxidase-like 2 (LOXL2) silencing in the tube formation of HUVECs, indicating that LOXL2 is implicated in DPSC-H-Exo-mediated angiogenesis. In accordance with these findings, as verified by Liu et al., exosomes isolated from hypoxic-preconditioned SHED (SHED-H-Exos) possessed positive effects on HUVEC angiogenesis by transferring let-7f-5p and miR-210-3p via the AGO1/VEGF and ephrinA3 signaling pathways, respectively [93].

In addition to hypoxic exosomes from dental MSCs, Han et al. disclosed that exosomes extracted from hypoxia-treated AMSCs encapsulated larger amounts of angiogenic-associated proteins, including VEGF, VEGFR2, VEGFR3, FGF, epidermal growth factor (EGF), and monocyte chemotactic protein 2, which exhibited a remarkably enhanced impact on the proliferation, migration, and vascular formation capabilities of HUVECs [94]. Of importance, the transcriptional regulator hypoxia-inducible factor-1α (HIF-1α) has been proposed as the most essential mediator of cellular adaptive responses to hypoxic conditions, thus orchestrating oxygen homeostasis [95]. It is noteworthy that exosomes secreted by DPSCs overexpressing HIF-1α (DPSC^HIF−1α^-Exos) potentiated capillary-like tubular structure formation in vitro and substantial vessel growth in vivo, which could be abrogated by Jagged1 antibodies [96]. In this context, DPSC^HIF−1α^-Exos shed new light on their pivotal roles in the angiogenic process of pulp regeneration via packaging Jagged1. Therefore, in the presence of hypoxia and associated angiogenic mediators, exosomes may serve as novel therapeutic tools to coordinate pulp angiogenesis in regenerative endodontic procedures. From the studies above, it can be concluded that alterations in the cell culture environment can significantly modify the active components transported by their originating exosomes, enabling them to perform various biological functions. In future research, the composition and function of exosomes should be further optimized by enhancing the cell culture environment to obtain desirable exosomes that meet the requirements for clinical applications in regenerative endodontics.

In addition to the various chemical factors mentioned earlier, low-intensity pulsed ultrasound (LIPUS) has been widely demonstrated to promote the proliferation, migration, differentiation, and homing capabilities of stem/progenitor cells [97,98]. Moreover, LIPUS can stimulate stem/progenitor cells to secrete more exosomes and affect their biological functions through miRNA-mediated mechanisms, such as improving their anti-inflammatory properties and osteogenic capacity [99,100,101,102] As reported by Zhu et al., in contrast with normal exosomes, exosomes derived from LIPUS-treated dental follicle stem cells (DFSC-L-Exos) could substantially strengthen the proliferation and osteogenic differentiation potential of DFSCs, indicative of promising applications for DFSC-LIPUS-Exos in the regeneration and repair of maxillofacial bone defects [103]. Importantly, in the field of RET, despite the fact that in vitro and in vivo experiments have revealed that LIPUS is capable of inducing the proliferation of odontoblasts and promoting the production of reparative dentin [104,105,106], the impact of exosomes obtained under LIPUS intervention on pulp–dentin complex regeneration has not been reported and deserves in-depth investigation. Furthermore, intense attention should be directed to the fact that low-level laser therapy (LLLT) can alter the redox state of cells and stimulate mRNA activation, DNA replication, and protein synthesis [107]. Accumulating evidence has indicated that LLLT notably enhanced the cell viability, proliferation potential, and odonto/osteogenic differentiation ability of dental-derived MSCs, such as SCAP [108], DPSCs [109], and SHED [110], thereby showing beneficial effects for promoting pulp regeneration. Specifically, according to Paschalidou et al., SHED exposed to laser irradiation at 8 J/cm^2^ energy fluence exhibited significantly upregulated expression of odonto/osteogenesis-relevant genes, such as *ALP* and *DSPP*, and pronounced deposition of mineral nodules, confirming that LLLT as an inductive factor may contribute to the regeneration of the pulp–dentin complex by promoting SHED biological processes [111]. Also, these results pave the way for narrowing the “therapeutic window” of LLLT-induced exosomes in RET. Another scientific proposal stemming from physical inductive conditions for exosome-based regenerative endodontics would be photobiomodulation therapy (PBMT). Marques et al. ranked PBMT alongside stem cells, scaffolds, and growth factors, referring to it as the fourth element of tissue engineering [112]. It has been shown that the employment of PBMT could modulate the inflammatory response and facilitate the migration and proliferation capabilities of dental-derived MSCs, such as SHED and DPSCs [113,114]. As documented by Moreira et al., PBMT at an energy density of 5 J/cm^2^ significantly improved the cell viability, proliferation, and migration capacities of SCAP incubated in chitosan/β-glycerophosphate hydrogels [115]. By evoking apical blood clots in rat molars, this group further illustrated that the application of PBMT efficiently induced cell transmigration through chitosan hydrogels and contributed to the regeneration of fully developed pulp tissue inside the root canals, which was characterized by abundant vascular networks and odontoblast-like cells with cellular protrusions penetrating the newly formed tubular dentin. Although PBMT has been verified to facilitate pulp–dentin complex regeneration by promoting cell recruitment, the effect of PBMT on exosomes and associated RET lacks definitive evidence and warrants profound research. Collectively, physical tissue engineering factors, such as LIPUS, LLLT, and PBMT, have emerged as novel strategies to improve the functions of exosomes and hold promise for advancing pulp–dentin complex regeneration.

### 4.3. Exosome Concentration

The concentration of exosomes can influence their biological availability and biostability in the application environment. Dedicated to elucidating the effect of the concentration of exosome-like vesicles derived from adipose tissue (AT-ELVs) on the biological functions of aorta endothelial cells, Dai et al. delineated that 10 and 50 μg/mL AT-ELVs could remarkably increase the number of migrated cells, with 50 μg/mL AT-ELVs being superior to 10 μg/mL AT-ELVs [116]. Notably, when the concentration of AT-ELVs was increased to 200 μg/mL, the migration capability of aortic endothelial cells was adversely inhibited. Similarly, CCK-8 experiments showed that 10 and 50 μg/mL AT-ELVs could enhance the proliferation of aortic endothelial cells in a dose-dependent manner. However, AT-ELVs at 200 μg/mL attenuated cellular proliferation ability, which was relatively lower than that of the control group. Therefore, this study indicates that low concentrations of AT-ELVs can promote the proliferation and migration of aortic endothelial cells, while on the contrary, high concentrations elicit inhibitory effects. Committed to clarifying the optimal concentration of HERS-ELVs to improve the functions of DPCs, Zhang et al. found that when the concentration of HERS-ELVs was 80 μg/mL, the proliferation ability of DPCs was the strongest and the number of migrated cells increased significantly [25]. However, when the concentration was elevated to 160 μg/mL, the proliferative and migratory potential of DPCs was weakened compared to the 80 μg/mL group but was still markedly higher than the control group. Notably, as the concentration increased to 240 μg/mL, the number of migrated cells was substantially reduced, exhibiting no significant difference from the control group. Collectively, the above results suggest that high concentrations of exosomes exert unfavorable effects instead of enhancing the biological functions of target cells. Considering this, in regenerative endodontic practice, selecting the appropriate exosome concentration plays a vital role in acquiring therapeutic efficacy. Exosomes with lower concentrations are susceptible to enzyme degradation or clearance by immune cells, incapable of initiating biological effects on target cells [117]. On the other hand, higher concentrations of exosomes may deliver excessive proteins or nucleic acids to target cells, causing cellular “overloading” that affects normal biological functions or induces undesirable immune responses and side effects [116]. In summary, before exploring clinical translation strategies for exosome-based pulp–dentin complex regeneration, particular attention should be paid to the optimal concentration of exosomes, ensuring biological functionality while maintaining safety.

### 4.4. Application Environment of Exosomes

To prevent exosomes from being diluted or degraded by body fluids, they must be transported to the site of action on suitable scaffold materials [118]. Consequently, the combinatory application of exosomes and scaffold materials has been recognized as a feasible and effective strategy in regenerative medicine to achieve sustained and controlled release of exosomes. Depending on their source, scaffold materials suitable for regenerative endodontics comprise natural and synthetic materials. Natural materials, such as chitosan and gelatin, possess favorable biocompatibility and bioactivity, while synthetic materials, such as poly(lactic-co-glycolic acid) (PLGA), show superior physical and mechanical properties as well as controllable degradation rates. In this regard, various types of polymer scaffolds have been exploited to encapsulate exosomes in the form of hydrogels, microspheres, and nanoparticles. Hydrogels are hydrophilic polymer materials that simulate the 3D network structures and biological properties of the extracellular matrix (ECM) [119]. Due to their superior mechanical, chemical, and biological properties, hydrogels are extensively considered the preferred materials for tissue engineering and biomedical applications, showing broad prospects for promoting the regeneration and repair of oral tissues, including the pulp–dentin complex, cementum, periodontal ligament, and alveolar bone [120,121,122]. Aiming at validating the usefulness and reliability of GelMA hydrogels for dental pulp regeneration, Khayat et al. encapsulated for the first time DPSCs and HUVECs in GelMA hydrogels and injected them into tooth root segments [123]. After subcutaneous transplantation for 8 weeks, DPSCs/HUVECs-encapsulated GelMA constructs reestablished well-organized and functional neovasculature containing red blood cells within highly cellularized and collagen-deposited dental pulp-like tissue. Odontoblast-like cells were aligned along the inner dentin surface with cellular protrusions infiltrating into the dentinal tubules, identifying that GelMA hydrogels are attractive for the development of clinically relevant scaffold materials in RET and hold promising applications to deliver exosomes. As a natural cationic polysaccharide, chitosan is obtained from the partial deacetylation of chitin and exhibits favorable biocompatibility, biodegradability, non-toxicity, and antibacterial activity [124]. Moreover, chitosan can be modified by introducing other functional groups to improve its physicochemical properties and biological functions [125]. Using rat hind limb ischemia models, Zhang et al. confirmed that chitosan hydrogel could control the release rate of exosomes and prolong their action time, thus achieving sustained release of exosomes [126]. Recently, in rat periodontitis models, Shen et al. reported that, in comparison with DPSC-Exo alone, the implantation of chitosan hydrogel incorporating DPSC-Exos significantly downregulated the protein levels of proinflammatory factors such as IL-23, IL-1α, IL-12, IL-1β, and TNF-α by inhibiting NF-κB and p38 MAPK signaling pathways [127]. In addition to the anti-inflammatory effect, chitosan hydrogel loaded with DPSC-Exos also possessed desirable immunomodulatory properties, which stimulated the polarization of macrophages from M1 to M2, correspondingly attenuating the inflammatory response and promoting periodontal regeneration. Given that eradicating root canal infection represents one of the essential bottlenecks to overcome for the clinical transformation of RET, the anti-inflammatory and immunomodulatory effects of chitosan hydrogel display immense potential and superiority in facilitating pulp–dentin complex regeneration. More importantly, compounding chitosan with other antibacterial materials would be advantageous to obtain a unique chitosan composite with comprehensive antibacterial properties for the elimination of residual infection and reinfection in root canals, which can provide a suitable immune microenvironment for regenerative endodontic procedures. Owing to their strong stability and ECM-like microstructure as well as their excellent biocompatibility, biodegradability, and injectability, self-assembling peptide-based hydrogels have become promising candidates for regenerative medicine [128]. According to Lan et al., who subcutaneously transplanted PuraMatrix peptide hydrogels encapsulating DPSC-Exos into nude mice for 30 days, vascular structure-enriched dental pulp tissue could be detected in the root canals, where collagen fibers and mineralized tissues were arranged in an orderly and longitudinal manner along the root canal wall [52]. PuraMatrix hydrogels are self-assembled peptide nanofiber scaffolds capable of permitting the controlled release of exosomes as the gels degrade. As a result, together with released exosomes, the degraded amino acids can be absorbed by target cells, thus creating favorable conditions for cellular adhesion, proliferation, and differentiation.

**Figure 4:**
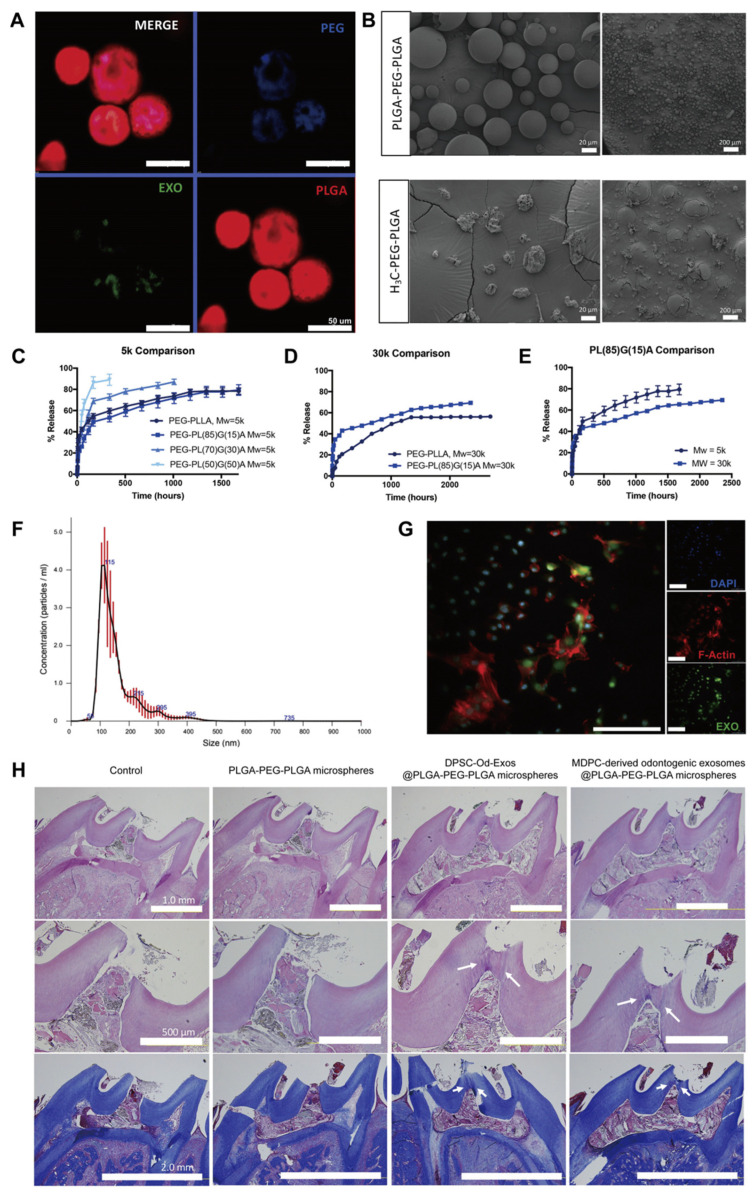
PLGA-PEG-PLGA polymeric microspheres encapsulating DPSC-Od-Exos facilitate tertiary dentin formation in vivo. (**A**,**B**) The structure of polymeric microspheres containing DPSC-Od-Exos. (**A**) As detected by confocal microscopy, the polymeric microspheres were mainly composed of PLGA segments (in red fluorescence), in which interior PEG domains (in blue fluorescence) formed porous structures. Notably, DPSC-Od-Exos (in green fluorescence) colocalized with PEG segments, thereby contributing to a “sphere-in-sphere” morphology. (**B**) In comparison with the deblock H_3_C-PEG-PLGA, which exhibited deflated spheres with rough surfaces, the triblock PLGA-PEG-PLGA was characterized by uniformly spherical structures with smooth surfaces. Scale bars are shown. (**C**–**G**) The release profile of DPSC-Od-Exos in vitro. (**C**–**E**) The release kinetics are reflective of the molecular weights and hydrophilicity of the triblock PLGA-PEG-PLGA. PL_85_G_15_A-PEG-PL_85_G_15_A (5 kDa) was acknowledged as the optimal chemical formulation due to the favorable sustained release of exosomes. (**F**) NTA analysis confirmed that the released DPSC-Od-Exos remained intact and undisturbed, which consequently presented a characteristic size and diameter distribution. (**G**) The released fluorescent DPSC-Od-Exos (in green fluorescence) could be taken up by DPSCs. Scale bar, 100 µm. (**H**) As illustrated by rat pulp capping models via HE staining (**top and middle rows**) and Masson’s trichrome staining (**bottom row**), the controlled release of DPSC-Od-Exos from PLGA-PEG-PLGA polymeric microspheres was beneficial for dentinogenesis at the pulp interface, which featured dentinal tubules and the accumulation of collagenous dentin matrix (arrows). Scale bars are shown. Reproduced with permission [55].

As discussed before, the physicochemical properties and biological functions of scaffold materials can be improved through chemical modification strategies or by incorporating microspheres or nanoparticles, which endow them with a variety of advantages in oral tissue engineering applications, such as better water solubility, adhesive potential, and antimicrobial properties, thereby reducing the reinfection of root canals and ensuring the stability and sustained release of exosomes [129]. As previously validated, the membrane surface of DPSC-Exos contains binding sites for type I collagen and fibronectin, and it is beneficial for the adherence of exosomes to matrix proteins such as type I collagen and fibronectin in the ECM through an integrin-mediated process [24]. In view of this, modifying conventional biomaterial scaffolds with collagen or fibronectin will increase the number of exosomes wrapped in the scaffold and promote their slow release. Microspheres are micro- and nano-sized spherical or sphere-like particles fabricated with biodegradable or absorbable polymer materials [130,131]. They display greater surface area and lower mass density as well as superior cell attachment, cell proliferation, drug absorption, and drug release kinetics, thus demonstrating distinct advantages as drug carriers and tissue engineering scaffolds. In this regard, Swanson et al. constructed the amphiphilic triblock copolymer microsphere PLGA-polyethylene glycol-PLGA (PLGA-PEG-PLGA) to encapsulate and sustain the release of DPSC-Od-Exos [55]. DPSC-Od-Exos@PLGA-PEG-PLGA exhibited uniform sphere-in-sphere structures with smooth surfaces (Figure 4). As revealed by the release kinetics of DPSC-Od-Exos in vitro, PL_85_G_15_A-PEG-PL_85_G_15_A (5 kDa) was considered the optimal chemical formulation due to the favorable controlled elution of exosomes. Moreover, the released exosomes maintained their integrity and biological activity and could be internalized by DPSCs. Encouragingly, when employed as pulp-capping materials in rat molars, the sustained release of DPSC-Od-Exos from PLGA-PEG-PLGA microspheres induced the migration and odontoblastic differentiation of resident endogenous pulp stem cells and enhanced the generation of tertiary dentin bridges at the pulp exposure sites, which were characterized by dentinal tubules and an abundant collagenous dentin matrix (Figure 4). These findings demonstrate that PLGA-PEG-PLGA polymeric microspheres represent novel and desirable vehicles for exosome delivery to foster complete pulp–dentin complex regeneration. Nanoparticles are nanoscale particles ranging from 10 to 100 nm in diameter. They are composed of natural or synthetic polymers and possess unique physicochemical properties and vigorous antibacterial and anti-biofilm activities [132]. According to Hussein et al., chitosan nanoparticle complexes have a stronger affinity and permeability to bacterial cell membranes, exerting robust antibacterial effects [133,134]. Additionally, they impose a significant scavenging impact on bacterial biofilms and can promote macrophage infiltration and periodontal ligament fibroblast migration, consequently inhibiting periapical tissue inflammation and inducing tissue repair. Accordingly, the synergistic application of chitosan nanoparticle complexes and exosomes for dental pulp regeneration holds promising prospects for root canal disinfection and ameliorating the regenerative microenvironment. Thus, selecting appropriate scaffold materials can improve the application environment of exosomes, achieve specificity in exosome delivery, and further enhance their therapeutic efficacy, thus creating favorable conditions for pulp–dentin complex regeneration.

## 5. Conclusions and Outlook

As our understanding deepens, the crucial role of exosomes in enhancing cell biological functions and promoting pulp–dentin complex regeneration has gained widespread attention. Compared with stem cell-based pulp regeneration, exosomes present the advantages of low acquisition costs, wide sources, good biocompatibility, and high safety [50]. In the field of regenerative endodontics, exosomes have shown great therapeutic potential and provide a strong impetus for the development and clinical translation of RET [135].

However, it is essential to note that exosome-based RET strategies still face multiple challenges and limitations. Firstly, the homogeneous production of exosomes represents one major barrier hindering their clinical translation and application. As mentioned earlier, the most commonly utilized methods for exosome separation in current times include ultracentrifugation, ultrafiltration, size-exclusion chromatography, immunoaffinity chromatography, polymer precipitation, and microfluidic techniques [30,136]. Although exosome isolation techniques have made significant progress in recent decades, there is still a lack of methods for absolutely precise isolation of exosomes due to their small particle size, overlapping particle size range, and similar morphology to other extracellular vesicles [137]. Secondly, the optimal source, concentration, parental cell culture environment, characterization, and modification methods of exosomes that are applicable to clinical treatment have yet to be elucidated. Therefore, there is an urgent need to standardize and optimize these influencing factors to ensure the optimal therapeutic efficacy of exosomes for their safe and effective use as pulp regeneration tools. Furthermore, the therapeutic effectiveness and safety of exosomes in pulp regeneration cannot be accurately evaluated, especially considering exosomes contain complex compositions, including proteins, lipids, and nucleic acids. In light of this, it is necessary to conduct compositional analysis such as RNA sequencing, proteomics, and metabolomics to determine whether their components are toxic or immunogenic. Last but not least, the prevalent in vivo research on exosome-induced regenerative endodontic procedures is constrained to animal experiments, and there is a paucity of robust clinical evidence. In addition, the molecular mechanisms underlying exosome-mediated intercellular communications; the adverse reactions that exosomes may induce; and the transport pathways, biological distribution, and metabolic kinetics of exosomes in the body remain to be elaborated [138]. Consequently, more carefully designed in vitro cell culture assays, in vivo animal experiments, and clinical trials simulating dental pulp physiological and pathological conditions are warranted in the future to guarantee the stability and safety of exosome treatment in clinical settings. It is believed that with the continuous advancement of exosome research and the promotion of multidisciplinary collaboration, exosomes will be clinically translated and applied in the field of regenerative endodontics, thus facilitating structural regeneration and functional reconstruction of the pulp–dentin complex and benefiting public oral health.

## References

[1] Ricucci D., Siqueira J.F., Abdelsayed R.A., Lio S.G., Rôças I.N. (2021). Bacterial invasion of pulp blood vessels in teeth with symptomatic irreversible pulpitis. J. Endod..

[2] Lee A.H., Cheung G.S., Wong M.C. (2012). Long-term outcome of primary non-surgical root canal treatment. Clin. Oral. Investig..

[3] Ricucci D., Siqueira J.F., Li Y., Tay F.R. (2019). Vital pulp therapy: Histopathology and histobacteriology-based guidelines to treat teeth with deep caries and pulp exposure. J. Dent..

[4] Cohenca N., Paranjpe A., Berg J. (2013). Vital pulp therapy. Dent. Clin. N. Am..

[5] Schwendicke F., Brouwer F., Schwendicke A., Paris S. (2016). Different materials for direct pulp capping: Systematic review and meta-analysis and trial sequential analysis. Clin. Oral. Investig..

[6] Ghoddusi J., Forghani M., Parisay I. (2014). New approaches in vital pulp therapy in permanent teeth. Iran. Endod. J..

[7] Cox C.F., Suzuki S. (1994). Re-evaluating pulp protection: Calcium hydroxide liners vs. cohesive hybridization. J. Am. Dent. Assoc..

[8] Asgary S., Eghbal M.J., Parirokh M., Ghanavati F., Rahimi H. (2008). A comparative study of histologic response to different pulp capping materials and a novel endodontic cement. Oral. Surg. Oral. Med. Oral. Pathol. Oral. Radiol. Endod..

[9] Giuroiu C.L., Căruntu I.D., Lozneanu L., Melian A., Vataman M., Andrian S. (2015). Dental pulp: Correspondences and contradictions between clinical and histological diagnosis. Biomed. Res. Int..

[10] Glickman G.N. (2009). AAE consensus conference on diagnostic terminology: Background and perspectives. J. Endod..

[11] Fonzar F., Fonzar A., Buttolo P., Worthington H.V., Esposito M. (2009). The prognosis of root canal therapy: A 10-year retrospective cohort study on 411 patients with 1175 endodontically treated teeth. Eur. J. Oral Implantol..

[12] Imura N., Pinheiro E.T., Gomes B.P., Zaia A.A., Ferraz C.C.R., Souza-Filho F.J. (2007). The outcome of endodontic treatment: A retrospective study of 2000 cases performed by a specialist. J. Endod..

[13] Shi X., Mao J., Liu Y. (2020). Pulp stem cells derived from human permanent and deciduous teeth: Biological characteristics and therapeutic applications. Stem Cells Transl. Med..

[14] Xie Z., Shen Z., Zhan P., Yang J., Huang Q., Huang S., Chen L., Lin Z. (2021). Functional dental pulp regeneration: Basic research and clinical translation. Int. J. Mol. Sci..

[15] Huang G.T., Yamaza T., Shea L.D., Djouad F., Kuhn N.Z., Tuan R.S., Shi S. (2010). Stem/progenitor cell-mediated de novo regeneration of dental pulp with newly deposited continuous layer of dentin in an in vivo model. Tissue Eng. Part A.

[16] Xuan K., Li B., Guo H., Sun W., Kou X., He X., Zhang Y., Sun J., Liu A., Liao L. (2018). Deciduous autologous tooth stem cells regenerate dental pulp after implantation into injured teeth. Sci. Transl. Med..

[17] de Cara S.P.H.M., Origassa C.S.T., de Sá Silva F., Moreira M.S.N.A., de Almeida D.C., Pedroni A.C.F., Carvalho G.L., Cury D.P., Câmara N.O.S., Marques M.M. (2019). Angiogenic properties of dental pulp stem cells conditioned medium on endothelial cells in vitro and in rodent orthotopic dental pulp regeneration. Heliyon..

[18] Murakami M., Hayashi Y., Iohara K., Osako Y., Hirose Y., Nakashima M. (2015). Trophic effects and regenerative potential of mobilized mesenchymal stem cells from bone marrow and adipose tissue as alternative cell sources for pulp/dentin regeneration. Cell Transplant..

[19] Yu S., Zhao Y., Fang T.J., Ge L. (2020). Effect of the soluble factors released by dental apical papilla-derived stem cells on the osteo/odontogenic, angiogenic, and neurogenic differentiation of dental pulp cells. Stem Cells Dev..

[20] Damania A., Jaiman D., Teotia A.K., Kumar A. (2018). Mesenchymal stromal cell-derived exosome-rich fractionated secretome confers a hepatoprotective effect in liver injury. Stem Cell Res. Ther..

[21] Kang T., Jones T.M., Naddell C., Bacanamwo M., Calvert J.W., Thompson W.E., Bond V.C., Chen Y.E., Liu D. (2016). Adipose-derived stem cells induce angiogenesis via microvesicle transport of miRNA-31. Stem Cells Transl. Med..

[22] Nakamura Y., Miyaki S., Ishitobi H., Matsuyama S., Nakasa T., Kamei N., Akimoto T., Higashi Y., Ochi M. (2015). Mesenchymal-stem-cell-derived exosomes accelerate skeletal muscle regeneration. FEBS Lett..

[23] Théry C., Zitvogel L., Amigorena S. (2002). Exosomes: Composition, biogenesis and function. Nat. Rev. Immunol..

[24] Huang C.C., Narayanan R., Alapati S., Ravindran S. (2016). Exosomes as biomimetic tools for stem cell differentiation: Applications in dental pulp tissue regeneration. Biomaterials.

[25] Zhang S., Yang Y., Jia S., Chen H., Duan Y., Li X., Wang S., Wang T., Lyu Y., Chen G. (2020). Exosome-like vesicles derived from Hertwig’s epithelial root sheath cells promote the regeneration of dentin-pulp tissue. Theranostics..

[26] Hammouda D.A., Mansour A.M., Saeed M.A., Zaher A.R., Grawish M.E. (2023). Stem cell-derived exosomes for dentin-pulp complex regeneration: A mini-review. Restor. Dent. Endod..

[27] Pan B.T., Johnstone R.M. (1983). Fate of the transferrin receptor during maturation of sheep reticulocytes in vitro: Selective externalization of the receptor. Cell..

[28] Johnstone R.M., Adam M., Hammond J.R., Orr L., Turbide C. (1987). Vesicle formation during re-ticulocyte maturation. Association of plasma membrane activities with released vesicles (exosomes). J. Biol. Chem..

[29] Tang Y., Zhou Y., Li H.J. (2021). Advances in mesenchymal stem cell exosomes: A review. Stem Cell Res. Ther..

[30] Hade M.D., Suire C.N., Suo Z. (2021). Mesenchymal stem cell-derived exosomes: Applications in regenerative medicine. Cells.

[31] Kalluri R., LeBleu V.S. (2020). The biology, function, and biomedical applications of exosomes. Science..

[32] Wei H., Chen Q., Lin L., Sha C., Li T., Liu Y., Yin X., Xu Y., Chen L., Gao W. (2021). Regulation of exosome production and cargo sorting. Int. J. Biol. Sci..

[33] Livshits M.A., Khomyakova E., Evtushenko E.G., Lazarev V.N., Kulemin N.A., Semina S.E., Generozov E.V., Govorun V.M. (2015). Isolation of exosomes by differential centrifugation: Theoretical analysis of a commonly used protocol. Sci. Rep..

[34] Zhang Y., Bi J., Huang J., Tang Y., Du S., Li P. (2020). Exosome: A review of its classification, isolation techniques, storage, diagnostic and targeted therapy applications. Int. J. Nanomed..

[35] Théry C., Witwer K.W., Aikawa E., Alcaraz M.J., Anderson J.D., Andriantsitohaina R., Antoniou A., Arab T., Archer F., Atkin-Smith G.K. (2018). Minimal information for studies of extracellular vesicles 2018 (MISEV2018): A position statement of the international society for extracellular vesicles and update of the MISEV2014 guidelines. J. Extracell Vesicles.

[36] Sahoo S., Losordo D.W. (2014). Exosomes and cardiac repair after myocardial infarction. Circ. Res..

[37] Vicencio J.M., Yellon D.M., Sivaraman V., Das D., Boi-Doku C., Arjun S., Zheng Y., Riquelme J.A., Kearney J., Sharma V. (2015). Plasma exosomes protect the myocardium from ischemia-reperfusion injury. J. Am. Coll. Cardiol..

[38] Zhang Z.G., Buller B., Chopp M. (2019). Exosomes—Beyond stem cells for restorative therapy in stroke and neurological injury. Nat. Rev. Neurol..

[39] Zagrean A.M., Hermann D.M., Opris I., Zagrean L., Popa-Wagner A. (2018). Multicellular crosstalk between exosomes and the neurovascular unit after cerebral ischemia. therapeutic implications. Front. Neurosci..

[40] Shi R., Jin Y., Hu W., Lian W., Cao C., Han S., Zhao S., Yuan H., Yang X., Shi J. (2020). Exosomes derived from mmu_circ_0000250-modified adipose-derived mesenchymal stem cells promote wound healing in diabetic mice by inducing miR-128-3p/SIRT1-mediated autophagy. Am. J. Physiol. Cell Physiol..

[41] Tan C.Y., Lai R.C., Wong W., Dan Y.Y., Lim S.-K., Ho H.K. (2014). Mesenchymal stem cell-derived exosomes promote hepatic regeneration in drug-induced liver injury models. Stem Cell Res. Ther..

[42] Yan Y., Jiang W., Tan Y., Zou S., Zhang H., Mao F., Gong A., Qian H., Xu W. (2017). hucMSC exosome-derived GPX1 is required for the eecovery of hepatic oxidant injury. Mol. Ther..

[43] Zhang S., Chu W.C., Lai R.C., Lim S.K., Hui J.H.P., Toh W.S. (2016). Exosomes derived from human embryonic mesenchymal stem cells promote osteochondral regeneration. Osteoarthr. Cartil..

[44] Furuta T., Miyaki S., Ishitobi H., Ogura T., Kato Y., Kamei N., Miyado K., Higashi Y., Ochi M. (2016). Mesenchymal stem cell-derived exosomes promote fracture healing in a mouse model. Stem Cells Transl. Med..

[45] Zhang J., Liu X., Li H., Chen C., Hu B., Niu X., Li Q., Zhao B., Xie Z., Wang Y. (2016). Exosomes/tricalcium phosphate combination scaffolds can enhance bone regeneration by activating the PI3K/Akt signaling pathway. Stem Cell Res. Ther..

[46] Tao S.-C., Yuan T., Zhang Y.-L., Yin W.-J., Guo S.-C., Zhang C.-Q. (2017). Exosomes derived from miR-140-5p-overexpressing human synovial mesenchymal stem cells enhance cartilage tissue regeneration and prevent osteoarthritis of the knee in a rat model. Theranostics.

[47] Shi X., Jiang N., Mao J., Luo D., Liu Y. (2021). Mesenchymal stem cell-derived exosomes for organ development and cell-free therapy. Nano Select..

[48] Zou J., Mao J., Shi X. (2022). Influencing factors of pulp-dentin complex regeneration and related biological strategies. J. Zhejiang Univ. (Med. Sci.).

[49] Yu S., Chen H., Gao B. (2020). Potential therapeutic effects of exosomes in regenerative endodontics. Arch. Oral. Biol..

[50] Ivica A., Ghayor C., Zehnder M., Valdec S., Weber F.E. (2020). Pulp-derived exosomes in a fibrin-based regenerative root filling material. J. Clin. Med..

[51] Li Z., Liang Y., Pan K., Li H., Yu M., Guo W., Chen G., Tian W. (2017). Schwann cells secrete extracellular vesicles to promote and maintain the proliferation and multipotency of hDPCs. Cell Prolif..

[52] Li J., Ju Y., Liu S., Fu Y., Zhao S. (2021). Exosomes derived from lipopolysaccharide-preconditioned human dental pulp stem cells regulate Schwann cell migration and differentiation. Connect. Tissue Res..

[53] Abdik H., Avsar Abdik E., Hızlı Deniz A.A., Taşlı P.N., Şahin F. (2019). A novel virtue in stem cell research: Exosomes and their role in differentiation. Adv. Exp. Med. Biol..

[54] Hu X., Zhong Y., Kong Y., Chen Y., Feng J., Zheng J. (2019). Lineage-specific exosomes promote the odontogenic differentiation of human dental pulp stem cells (DPSCs) through TGFβ1/smads signaling pathway via transfer of microRNAs. Stem Cell Res. Ther..

[55] Swanson W.B., Gong T., Zhang Z., Eberle M., Niemann D., Dong R., Rambhia K.J., Ma P.X. (2020). Controlled release of odontogenic exosomes from a biodegradable vehicle mediates dentinogenesis as a novel biomimetic pulp capping therapy. J. Control Release..

[56] Zhuang X., Ji L., Jiang H., Liu Y., Liu X., Bi J., Zhao W., Ding Z., Chen X. (2020). Exosomes derived from stem cells from the apical papilla promote dentine-pulp complex regeneration by inducing specific dentinogenesis. Stem Cells Int..

[57] Li B., Xian X., Lin X., Huang L., Liang A., Jiang H., Gong Q. (2022). Hypoxia alters the proteome profile and enhances the angiogenic potential of dental pulp stem cell-derived exosomes. Biomolecules..

[58] Zhang Z., Shuai Y., Zhou F., Yin J., Hu J., Guo S., Wang Y., Liu W. (2020). PDLSCs regulate angiogenesis of periodontal ligaments via VEGF transferred by exosomes in periodontitis. Int. J. Med. Sci..

[59] Xian X., Gong Q., Li C., Guo B., Jiang H. (2018). Exosomes with highly angiogenic potential for possible use in pulp regeneration. J. Endod..

[60] Wu M., Liu X., Li Z., Huang X., Guo H., Guo X., Yang X., Li B., Xuan K., Jin Y. (2021). SHED aggregate exosomes shuttled miR-26a promote angiogenesis in pulp regeneration via TGF-β/SMAD2/3 signalling. Cell Prolif..

[61] Chai Y., Jiang X., Ito Y., Bringas P., Han J., Rowitch D.H., Soriano P., McMahon A.P., Sucov H.M. (2000). Fate of the mammalian cranial neural crest during tooth and mandibular morphogenesis. Development.

[62] Luo L., He Y., Wang X., Key B., Lee B.H., Li H., Ye Q. (2018). Potential roles of dental pulp stem cells in neural regeneration and repair. Stem Cells Int..

[63] Venugopal C., Rai K.S., Pinnelli V.B., Kutty B.M., Dhanushkodi A. (2018). Neuroprotection by human dental pulp mesenchymal stem cells: From billions to nano. Curr. Gene Ther..

[64] Mao Q., Nguyen P.D., Shanti R.M., Shi S., Shakoori P., Zhang Q., Le A.D. (2019). Gingiva-derived mesenchymal stem cell extracellular vesicles activate schwann cell repair phenotype and promote nerve regeneration. Tissue Eng. Part A.

[65] Jarmalavičiūtė A., Tunaitis V., Pivoraitė U., Venalis A., Pivoriūnas A. (2015). Exosomes from dental pulp stem cells rescue human dopaminergic neurons from 6-hydroxy-dopamine-induced apoptosis. Cytotherapy.

[66] Narayanan K., Kumar S., Padmanabhan P., Gulyas B., Wan A.C., Rajendran V.M. (2018). Lineage-specific exosomes could override extracellular matrix mediated human mesenchymal stem cell differentiation. Biomaterials.

[67] Gronthos S., Brahim J., Li W., Fisher L.W., Cherman N., Boyde A., DenBesten P., Robey P.G., Shi S. (2002). Stem cell properties of human dental pulp stem cells. J. Dent. Res..

[68] Grottkau B.E., Purudappa P.P., Lin Y.F. (2010). Multilineage differentiation of dental pulp stem cells from green fluorescent protein transgenic mice. Int. J. Oral. Sci..

[69] Kim S., Shin S.J., Song Y., Kim E. (2015). In vivo experiments with dental pulp stem cells for pulp-dentin complex regeneration. Mediators Inflamm..

[70] Martinez Saez D., Sasaki R.T., Neves A.D., Da Silva M.C. (2016). Stem cells from human exfoliated deciduous teeth: A growing literature. Cells Tissues Organs..

[71] Guo H., Zhao W., Liu A., Wu M., Shuai Y., Li B., Huang X., Liu X., Yang X., Guo X. (2020). SHED promote angiogenesis in stem cell-mediated dental pulp regeneration. Biochem. Biophys. Res. Commun..

[72] Yuan X., Yuan Z., Wang Y., Wan Z., Wang X., Yu S., Han J., Huang J., Xiong C., Ge L. (2022). Vascularized pulp regeneration via injecting simvastatin functionalized GelMA cryogel microspheres loaded with stem cells from human exfoliated deciduous teeth. Mater. Today Bio..

[73] Vu H.T., Han M.-R., Lee J.-H., Kim J.-S., Shin J.-S., Yoon J.-Y., Park J.-H., Dashnyam K., Knowles J.C., Lee H.-H. (2022). Investigating the effects of conditioned media from stem cells of human exfoliated deciduous teeth on dental pulp stem cells. Biomedicines.

[74] Sonoyama W., Liu Y., Fang D., Yamaza T., Seo B.-M., Zhang C., Liu H., Gronthos S., Wang C.-Y., Shi S. (2006). Mesenchymal stem cell-mediated functional tooth regeneration in swine. PLoS ONE.

[75] Na S., Zhang H., Huang F., Wang W., Ding Y., Li D., Jin Y. (2016). Regeneration of dental pulp/dentine complex with a three-dimensional and scaffold-free stem-cell sheet-derived pellet. J. Tissue Eng. Regen. Med..

[76] Bi F., Guo W.H. (2022). Advances in research on Hertwig’s epithelial root sheath cells. J. Kunming Med. Univ..

[77] Huang C.H., Ma Y.Y., Ren L., Cai Q., Chen R., Fu Q. (2018). Conditioned media and exosomes from rat adipose-derived mesenchymal stem cells enhance bone regeneration: A study in vitro. Chin. J. Stomatol. Res. (Electron. Ed.).

[78] Song Z.J., Huang C.H., Ren L., Cai Q., Chen R., Fu Q. (2017). The effect of murine adipose-derived stem cells exosomes on hypoxia induced osteocytes apoptosis. Chin. J. Stomatol. Res. (Electron. Ed.).

[79] Zhao H., Shang Q., Pan Z., Bai Y., Li Z., Zhang H., Zhang Q., Guo C., Zhang L., Wang Q. (2018). Exosomes from adipose-derived stem cells attenuate adipose inflammation and obesity through polarizing M2 macrophages and beiging in white adipose tissue. Diabetes.

[80] Huang C., Bao L., Lin T., Lu Y., Wu Y. (2020). Proliferation and odontogenic differentiation of human umbilical cord mesenchymal stem cells and human dental pulp cells co-cultured in hydrogel. Arch. Oral. Biol..

[81] Zhu Z., Zhang Y., Zhang Y., Zhang H., Liu W., Zhang N., Zhang X., Zhou G., Wu L., Hua K. (2019). Exosomes derived from human umbilical cord mesenchymal stem cells accelerate growth of VK2 vaginal epithelial cells through MicroRNAs in vitro. Hum. Reprod..

[82] Shi Y., Yang Y., Guo Q., Gao Q., Ding Y., Wang H., Xu W., Yu B., Wang M., Zhao Y. (2019). Exosomes derived from human umbilical cord mesenchymal stem cells promote fibroblast-to-myofibroblast differentiation in inflammatory environments and benefit cardioprotective effects. Stem Cells Dev..

[83] Chen Y., Yu Y., Chen L., Ye L., Cui J., Sun Q., Li K., Li Z., Liu L. (2015). Human umbilical cord mesenchymal stem cells: A new therapeutic option for tooth regeneration. Stem Cells Int..

[84] Brizuela C., Meza G., Urrejola D., Quezada M., Concha G., Ramírez V., Angelopoulos I., Cadiz M., Tapia-Limonchi R., Khoury M. (2020). Cell-based regenerative endodontics for treatment of periapical lesions: A randomized, controlled phase I/II clinical trial. J. Dent. Res..

[85] Huang X., Qiu W., Pan Y., Li J., Chen Z., Zhang K., Luo Y., Wu B., Xu W. (2021). Exosomes from LPS-stimulated hDPSCs activated the angiogenic potential of HUVECs in vitro. Stem Cells Int..

[86] Qiao X., Tang J., Dou L., Yang S., Sun Y., Mao H., Yang D. (2023). Dental pulp stem cell-derived exosomes regulate anti-inflammatory and osteogenesis in periodontal ligament stem cells and promote the repair of experimental periodontitis in rats. Int. J. Nanomed..

[87] Wang M., Li J., Ye Y., He S., Song J. (2020). SHED-derived conditioned exosomes enhance the osteogenic differentiation of PDLSCs via Wnt and BMP signaling in vitro. Differentiation.

[88] Faruqu F.N., Zhou S., Sami N., Gheidari F., Lu H., Al-Jamal K.T. (2020). Three-dimensional culture of dental pulp pluripotent-like stem cells (DPPSCs) enhances Nanog expression and provides a serum-free condition for exosome isolation. FASEB Bioadv..

[89] Zhang Q. (2019). The Study of Exosomes Derived from Aggregates of Stem Cells from Human Exfoliated Deciduous Teeth on Dental Pulp Regeneration. PhD Dissertation.

[90] Liu D., Shi B., Zhou W., Tao G. (2023). Exosomes from hypoxia-conditioned apical papilla stem cells accelerate angiogenesis in vitro through Notch/JAG1/VEGF signaling. Tissue Cell..

[91] Dorayappan K.D.P., Wanner R., Wallbillich J.J., Saini U., Zingarelli R., Suarez A.A., Cohn D.E., Selvendiran K. (2018). Hypoxia-induced exosomes contribute to a more aggressive and chemoresistant ovarian cancer phenotype: A novel mechanism linking STAT3/Rab proteins. Oncogene.

[92] Liu W., Li L., Rong Y., Qian D., Chen J., Zhou Z., Luo Y., Jiang D., Cheng L., Zhao S. (2020). Hypoxic mesenchymal stem cell-derived exosomes promote bone fracture healing by the transfer of miR-126. Acta Biomater..

[93] Liu P., Qin L., Liu C., Mi J., Zhang Q., Wang S., Zhuang D., Xu Q., Chen W., Guo J. (2022). Exosomes derived from hypoxia-conditioned stem cells of human deciduous exfoliated teeth enhance angiogenesis via the transfer of let-7f-5p and miR-210-3p. Front. Cell Dev. Biol..

[94] Han Y., Ren J., Bai Y., Pei X., Han Y. (2019). Exosomes from hypoxia-treated human adipose-derived mesenchymal stem cells enhance angiogenesis through VEGF/VEGF-R. Int. J. Biochem. Cell Biol..

[95] McGettrick A.F., O’Neill L.A.J. (2020). The role of HIF in immunity and inflammation. Cell Metab..

[96] Gonzalez-King H., García N.A., Ontoria-Oviedo I., Ciria M., Montero J.A., Sepúlveda P. (2017). Hypoxia inducible factor-1alpha potentiates jagged 1-mediated angiogenesis by mesenchymal stem cell-derived exosomes. Stem Cells..

[97] Padilla F., Puts R., Vico L., Raum K. (2014). Stimulation of bone repair with ultrasound: A review of the possible mechanic effects. Ultrasonics.

[98] Miller D.L., Smith N.B., Bailey M.R., Czarnota G.J., Hynynen K., Makin I.R.S. (2012). Overview of therapeutic ultrasound applications and safety considerations. J. Ultrasound Med..

[99] He Y.-F., Wang X.-L., Deng S.-P., Wang Y.-L., Huang Q.-Q., Lin S., Lyu G.-R. (2023). Latest progress in low-intensity pulsed ultrasound for studying exosomes derived from stem/progenitor cells. Front Endocrinol..

[100] Chen D., Xiang M., Gong Y., Xu L., Zhang T., He Y., Zhou M., Xin L., Li J., Song J. (2019). LIPUS promotes FOXO1 accumulation by downregulating miR-182 to enhance osteogenic differentiation in hPDLCs. Biochimie.

[101] Costa V., Carina V., Conigliaro A., Raimondi L., De Luca A., Bellavia D., Salamanna F., Setti S., Alessandro R., Fini M. (2019). MiR-31-5p is a LIPUS-mechanosensitive microRNA that targets HIF-1α signaling and cytoskeletal proteins. Int. J. Mol. Sci..

[102] Li X., Zhong Y., Zhou W., Song Y., Li W., Jin Q., Gao T., Zhang L., Xie M. (2023). Low-intensity pulsed ultrasound (LIPUS) enhances the anti-inflammatory effects of bone marrow mesenchymal stem cells (BMSCs)-derived extracellular vesicles. Cell Mol. Biol. Lett..

[103] Zhu M.Y. (2020). Effect and the Possible Mechanism of LIPUS-Stimulated Human Dental Follicle Stem Cell-Derived Exosomes on the Proliferation and Differentiation of hDFSCs. Master’s Thesis.

[104] Al-Daghreer S., Doschak M., Sloan A.J., Major P.W., Heo G., Scurtescu C., Tsui Y.Y., El-Bialy T. (2012). Long term effect of low intensity pulsed ultrasound on a human tooth slice organ culture. Arch. Oral. Biol..

[105] Wang F., Li Y., Yang Z., Lu K., Zuo J., Zhou Z. (2017). Effect of low-intensity pulsed ultrasound on a rat model of dentin-dental pulp injury and repair. Ultrasound Med. Biol..

[106] Zuo J., Zhen J., Wang F., Li Y., Zhou Z. (2018). Effect of Low-Intensity Pulsed Ultrasound on the Expression of Calcium Ion Transport-Related Proteins during Tertiary Dentin For-mation. Ultrasound Med. Biol..

[107] Asnaashari M., Safavi N. (2013). Application of low level lasers in dentistry (endodontic). J. Lasers Med. Sci..

[108] Gutiérrez D., Rouabhia M., Ortiz J., Gaviria D., Alfonso C., Muñoz A., Inostroza C. (2021). Low-level laser irradiation promotes proliferation and differentiation on apical papilla stem cells. J. Lasers Med. Sci..

[109] Tabatabaei F.S., Torshabi M., Mojahedi Nasab M., Khosraviani K., Khojasteh A. (2015). Effect of low-level diode laser on proliferation and osteogenic differentiation of dental pulp stem cells. Laser Phys..

[110] Ginani F., Soares D.M., de Oliveira Rocha H.A., de Souza L.B., Barboza C.A.G. (2018). Low-level laser irradiation induces in vitro proliferation of stem cells from human exfoliated deciduous teeth. Lasers Med. Sci..

[111] Paschalidou M., Athanasiadou E., Arapostathis K., Kotsanos N., Koidis P.T., Bakopoulou A., Theocharidou A. (2020). Biological effects of low-level laser irradiation (LLLI) on stem cells from human exfoliated deciduous teeth (SHED). Clin. Oral. Investig..

[112] Marques M.M., Diniz I.M., de Cara S.P., Pedroni A.C.F., Abe G.L., D’Almeida-Couto R.S., Lima P.L.V., Tedesco T.K., Moreira M.S. (2016). Photobiomodulation of dental derived mesenchymal stem cells: A systematic review. Photomed. Laser Surg..

[113] Staffoli S., Romeo U., Amorim R.N.S., Migliau G., Palaia G., Resende L., Polimeni A. (2017). The effects of low level laser irradiation on proliferation of human dental pulp: A narrative review. Clin. Ter..

[114] Ferreira L.S., Diniz I.M.A., Maranduba C.M.S., Miyagi S.P.H., Rodrigues M.F.S.D., Moura-Netto C., Marques M.M. (2019). Short-term evaluation of photobiomodulation therapy on the proliferation and undifferentiated status of dental pulp stem cells. Lasers Med. Sci..

[115] Moreira M.S., Sarra G., Carvalho G.L., Gonçalves F., Caballero-Flores H.V., Pedroni A.C.F., Lascala C.A., Catalani L.H., Marques M.M. (2021). Physical and biological properties of a chitosan hydrogel scaffold associated to photobiomodulation therapy for dental pulp regeneration: An in vitro and in vivo study. Biomed. Res. Int..

[116] Dai M., Yu M., Zhang Y., Tian W. (2017). Exosome-like vesicles derived from adipose tissue provide biochemical cues for adipose tissue regeneration. Tissue Eng. Part A.

[117] Shi J., Teo K.Y.W., Zhang S., Lai R.C., Rosa V., Tong H.J., Duggal M.S., Lim S.K., Toh W.S. (2023). Mesenchymal stromal cell exosomes enhance dental pulp cell functions and promote pulp-dentin regeneration. Biomater. Biosyst..

[118] Namjoynik A., Islam M.A., Islam M. (2023). Evaluating the efficacy of human dental pulp stem cells and scaffold combination for bone regeneration in animal models: A systematic review and meta-analysis. Stem Cell Res. Ther..

[119] Mantha S., Pillai S., Khayambashi P., Upadhyay A., Zhang Y., Tao O., Pham H.M., Tran S.D. (2019). Smart hydrogels in tissue engineering and regenerative medicine. Materials.

[120] Moussa D.G., Aparicio C. (2019). Present and future of tissue engineering scaffolds for dentin-pulp complex regeneration. J. Tissue Eng. Regen. Med..

[121] Huang M., Huang Y., Liu H., Tang Z., Chen Y., Huang Z., Xu S., Du J., Jia B. (2022). Hydrogels for the treatment of oral and maxillofacial diseases: Current research, challenges, and future directions. Biomater. Sci..

[122] Zheng L., Liu Y., Jiang L., Wang X., Chen Y., Li L., Song M., Zhang H., Zhang Y.S., Zhang X. (2023). Injectable decellularized dental pulp matrix-functionalized hydrogel microspheres for endodontic regeneration. Acta Biomater..

[123] Khayat A., Monteiro N., Smith E.E., Pagni S., Zhang W., Khademhosseini A., Yelick P. (2017). GelMA-encapsulated hDPSCs and HUVECs for dental pulp regeneration. J. Dent. Res..

[124] Khattak S., Wahid F., Liu L.P., Jia S.-R., Chu L.-Q., Xie Y.-Y., Li Z.-X., Zhong C. (2019). Applications of cellulose and chitin/chitosan derivatives and composites as antibacterial materials: Current state and perspectives. Appl. Microbiol. Biotechnol..

[125] Wang Y., Zou J., Cai M., Wang Y., Mao J., Shi X. (2023). Applicatoin of chitosan-based hydrogel in oral tissue engineering. J. Cent. South Univ. (Med. Sci.).

[126] Zhang K., Zhao X., Chen X., Wei Y., Du W., Wang Y., Liu L., Zhao W., Han Z., Kong D. (2018). Enhanced therapeutic effects of mesenchymal stem cell-derived exosomes with an injectable hydrogel for hindlimb ischemia treatment. ACS Appl. Mater. Interfaces.

[127] Shen Z., Kuang S., Zhang Y., Yang M., Qin W., Shi X., Lin Z. (2020). Chitosan hydrogel incorporated with dental pulp stem cell-derived exosomes alleviates periodontitis in mice via a macrophage-dependent mechanism. Bioact. Mater..

[128] Li J., Xing R., Bai S., Yan X. (2019). Recent advances of self-assembling peptide-based hydrogels for biomedical applications. Soft Matter..

[129] Ghasemi-Mobarakeh L., Prabhakaran M.P., Tian L., Shamirzaei-Jeshvaghani E., Dehghani L., Ramakrishna S. (2015). Structural properties of scaffolds: Crucial parameters towards stem cells differentiation. World J. Stem Cells..

[130] Hossain K.M.Z., Patel U., Ahmed I. (2015). Development of microspheres for biomedical applications: A review. Prog. Biomater..

[131] Bi Y.G., Lin Z.T., Deng S.T. (2019). Fabrication and characterization of hydroxyapatite/sodium alginate/chitosan composite microspheres for drug delivery and bone tissue engineering. Mater. Sci. Eng. C Mater. Biol. Appl..

[132] Li X., Chen D., Xie S. (2021). Current progress and prospects of organic nanoparticles against bacterial biofilm. Adv. Colloid. Interface Sci..

[133] Hussein H., Kishen A. (2020). Antibiofilm and immune response of engineered bioactive nanoparticles for endodontic disinfection. J. Clin. Med..

[134] Hussein H., Kishen A. (2021). Proteomic profiling reveals engineered chitosan nanoparticles mediated cellular crosstalk and immunomodulation for therapeutic application in apical periodontitis. Bioact. Mater..

[135] Yu Q. (2021). Progress in the diagnosis and treatment strategies of caries-derived dental pulp diseases. Chin. J. Stomatol..

[136] Zou J., Xia H., Jiang Q., Su Z., Wen S., Liang Z., Ouyang Y., Liu J., Zhang Z., Chen D. (2023). Exosomes derived from odontogenic stem cells: Its role in the dentin-pulp complex. Regen. Ther..

[137] Zarà M., Amadio P., Campodonico J., Sandrini L., Barbieri S.S. (2020). Exosomes in cardiovascular diseases. Diagnostics.

[138] Chen Y., Yang X.T., Ma Y., Yang B., Tian W.D. (2021). Exosomes-based strategies for dental pulp regeneration. Zhonghua Kou Qiang Yi Xue Za Zhi.

